# Across-country genetic and genomic analyses of foot score traits in American and Australian Angus cattle

**DOI:** 10.1186/s12711-023-00850-x

**Published:** 2023-11-02

**Authors:** Amanda B. Alvarenga, Kelli J. Retallick, Andre Garcia, Stephen P. Miller, Andrew Byrne, Hinayah R. Oliveira, Luiz F. Brito

**Affiliations:** 1 Department of Animal Sciences, Purdue University, 270 South Russell Street, West Lafayette, IN 47907 USA; 2 American Angus Association, Angus Genetics Inc., 3201 Frederick Avenue, Saint Joseph, MO 64506 USA; 3 AGBU, a Joint Venture of NSW Department of Primary Industries and University of New England, Armidale, NSW 2351 Australia; 4 Angus Australia, 86 Glen Innes Road, Armidale, NSW 2350 Australia

## Abstract

**Background:**

Hoof structure and health are essential for the welfare and productivity of beef cattle. Therefore, we assessed the genetic and genomic background of foot score traits in American (US) and Australian (AU) Angus cattle and investigated the feasibility of performing genomic evaluations combining data for foot score traits recorded in US and AU Angus cattle. The traits evaluated were foot angle (FA) and claw set (CS). In total, 109,294 and ~ 1.12 million animals had phenotypic and genomic information, respectively. Four sets of analyses were performed: (1) genomic connectedness between US and AU Angus cattle populations and population structure, (2) estimation of genetic parameters, (3) single-step genomic prediction of breeding values, and (4) single-step genome-wide association studies for FA and CS.

**Results:**

There was no clear genetic differentiation between US and AU Angus populations. Similar heritability estimates (FA: 0.22–0.24 and CS: 0.22–0.27) and moderate-to-high genetic correlations between US and AU foot scores (FA: 0.61 and CS: 0.76) were obtained. A joint-genomic prediction using data from both populations outperformed within-country genomic evaluations. A genomic prediction model considering US and AU datasets as a single population performed similarly to the scenario accounting for genotype-by-environment interactions (i.e., multiple-trait model considering US and AU records as different traits), even though the genetic correlations between countries were lower than 0.80. Common significant genomic regions were observed between US and AU for FA and CS. Significant single nucleotide polymorphisms were identified on the *Bos taurus* (BTA) chromosomes BTA1, BTA5, BTA11, BTA13, BTA19, BTA20, and BTA23. The candidate genes identified were primarily from growth factor gene families, including *FGF12* and *GDF5*, which were previously associated with bone structure and repair.

**Conclusions:**

This study presents comprehensive population structure and genetic and genomic analyses of foot scores in US and AU Angus cattle populations, which are essential for optimizing the implementation of genomic selection for improved foot scores in Angus cattle breeding programs. We have also identified candidate genes associated with foot scores in the largest Angus cattle populations in the world and made recommendations for genomic evaluations for improved foot score traits in the US and AU.

**Supplementary Information:**

The online version contains supplementary material available at 10.1186/s12711-023-00850-x.

## Background

Lowly-to-moderately heritable traits (e.g., structural problems and functional traits) in livestock are substantially affected by environmental factors. Structural problems have been a concern in both beef and dairy cattle breeding schemes due to their impact on animal welfare, increased complexity in daily management (e.g., lame animals can take longer to go through the chute), and economic losses due to costs of treatments and negative impact on production and fertility [1, 2]. Structural problems can also be known as “structural soundness” or “conformation”, which assess the body structure of an animal, e.g., feet and leg conformation. In North American Angus, it has been estimated that 6% of the cows are culled due to structural problems by having a direct impact on cow’s longevity [3]. Furthermore, North American and Australian Angus producers have ranked structural problems (e.g., feet conformation) within their top-three breeding goals [4, 5]. Foot angle (FA) and claw set (CS) are two key indicators of structural problems used in beef cattle breeding [6, 7].

Over the past decades, advancements in reproductive technologies (e.g., artificial insemination, sexed semen, and embryo transfer) have enabled higher-genetic-merit animals to produce more offspring under vastly different management systems and geographical regions [8]. In this context, the evaluation of genotype-by-environment interactions (GxE; [9]) becomes essential considering the existing exchange of genetic material across countries, in which the animal’s offspring performance is not necessarily similar across environments. Consequently, the welfare of animals that are poorly adapted to certain environments or management systems could be compromised, which results in losses of revenue. GxE have been acknowledged in breeding programs even prior to the implementation of genomic selection [10, 11]. Studies in beef cattle populations have reported different levels of GxE and changes in ranking of breeding animals across regions within a country [12, 13], in which the GxE can be explained by several environmental parameters. Characterization of GxE can be done, for instance, by using the temperature–humidity index (THI; e.g., using the American Angus dataset [12]) or by modelling broader environmental gradients such as macro-environments (i.e., Victoria versus Queensland using the Australian Angus dataset [13]). The magnitude of GxE can vary within the same population, depending on the trait being analyzed [13]. If an GxE is present, it can be accounted for by fitting multiple-trait models (categorical environmental levels, e.g., countries) or random regression models—reaction norms (continuous environmental gradients, e.g., THI [14]). In this context, Hayes et al. [14] indicated that incorporating GxE into the genomic prediction of breeding values could result in more accurate rankings of breeding candidates for specific environmental conditions, and therefore, faster genetic progress for the traits of interest.

Across-country genetic evaluations in beef cattle have been implemented or are under investigation in various regions around the world [15–20]. An international genetic evaluation could contribute to increasing genetic progress of smaller populations in single countries [17], as it might result in higher selection intensity and more accurate genomic estimated breeding values (GEBV) when larger training populations are available for genomic prediction of breeding values. The pedigree link and the performance of animals’ progeny across environments (e.g., countries) are key limitations for joint genetic evaluations [17–21]. Strong across-environment genetic connections are paramount for a proper estimation of the genetic correlation across environments, which is also needed for genomic evaluations [18, 21]. However, with the implementation of genomic selection, when pedigree information is not available (or limited), GEBV can be calculated for individuals across environments by multiplying the gene content (allele genotype information) by the specific-environment single nucleotide polymorphism (SNP) effect (when non-additive genetic effects are disregarded [14]).

Joint genomic evaluations can be beneficial to beef cattle breeding programs as they provide an opportunity to increase the size of reference populations, especially for novel or lowly-heritable traits, and consequently increase the accuracy of GEBV without significant additional costs to producers [18–23]. Considering the importance of foot score traits for Angus cattle populations, the primary objectives of this study were to assess the genetic and genomic background of foot scores in American (US) and Australian (AU) Angus cattle and investigate the feasibility of performing a joint genomic evaluation for FA and CS based on alternative genomic prediction scenarios. The specific objectives were to: (1) assess population structure and genomic connectedness between the US and AU Angus cattle populations using consistency of gametic phase, linkage disequilibrium, principal component analysis, and admixture, (2) estimate genetic parameters, (3) perform single-step genomic prediction of breeding values based on alternative scenarios, and (4) conduct single-step genome-wide association studies (ssGWAS) and post-ssGWAS analyses for FA and CS.

## Methods

### Datasets

#### Foot scoring system

**Fig. 1:**
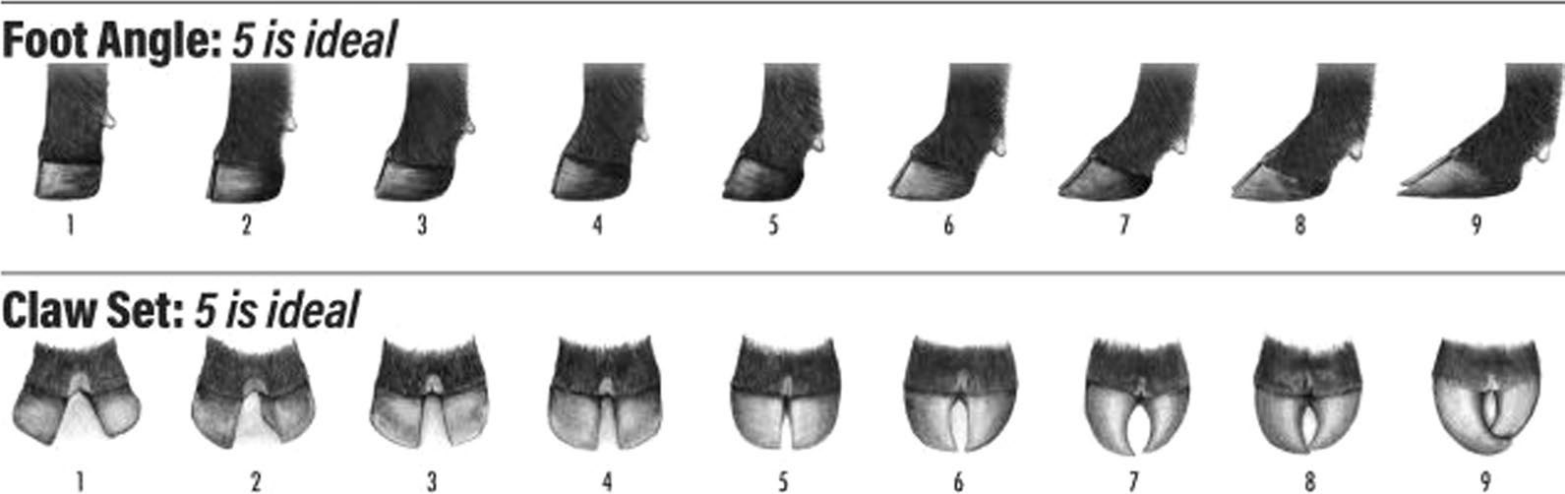
Foot score guidelines by the American Angus Association and Angus Australia (Picture source: American Angus Association [7])

Both the American Angus Association (AAA; Saint Joseph, MO, USA) and Angus Australia (AAU; Armidale, NSW, Australia) use similar scoring systems: FA (on a one-to-nine scale) and CS (on a one-to-nine scale). Both FA and CS are subjective measurements, and the scoring system is presented in Fig. 1, in which 5 is the optimal score for FA and CS [7]. For FA, a 5-score animal would have a 45-degree set to its pastern with acceptable heal depth. For CS, a score of 5 indicates a straight and symmetrical claw. Phenotypic recording is done on animals at yearling (1-year old) age and/or older animals. Collecting repeated measurements is encouraged (but still limited) and should be performed prior to hoof trimming.

The AAA recommends that the handlers record FA and CS on the worst foot of each animal when contemporary animals are being handled with a single handler recording all scores within a contemporary group (CG). Foot scores from AAU animals are mainly recorded by trained technicians and two scores are available for each animal and metric: one for the front and on for the rear leg. However, only the worst scored leg in the AAU was used for this study to mimic the AAA dataset. More details about the recording performed by the AAA and AAU can be found at the AAA website [7] and www.angusaustralia.com.au/education/breeding-and-genetics/collection-guidelines-for-angus-research/collecting-mature-cow-structural-soundness-scores/ (accessed 27 July 2022). Foot score CG were formed by concatenating management factors, which differed between AAA and AAU. The CG from AAA was defined by the concatenation of sex, weaning and yearling information [herd, measurement date, and management code (e.g., creep feeding system)]; diet type (i.e., pasture, low concentrate diet < 50%, and high concentrate diet > 50%), and herd and date when the foot scores were collected. The CG for AAU was formed by concatenating sex, weaning and yearling management groups, management group defined at the time of scoring the traits, and in which leg the score was collected (i.e., front or rear; [24]).

#### Phenotypic datasets

**Fig. 2:**
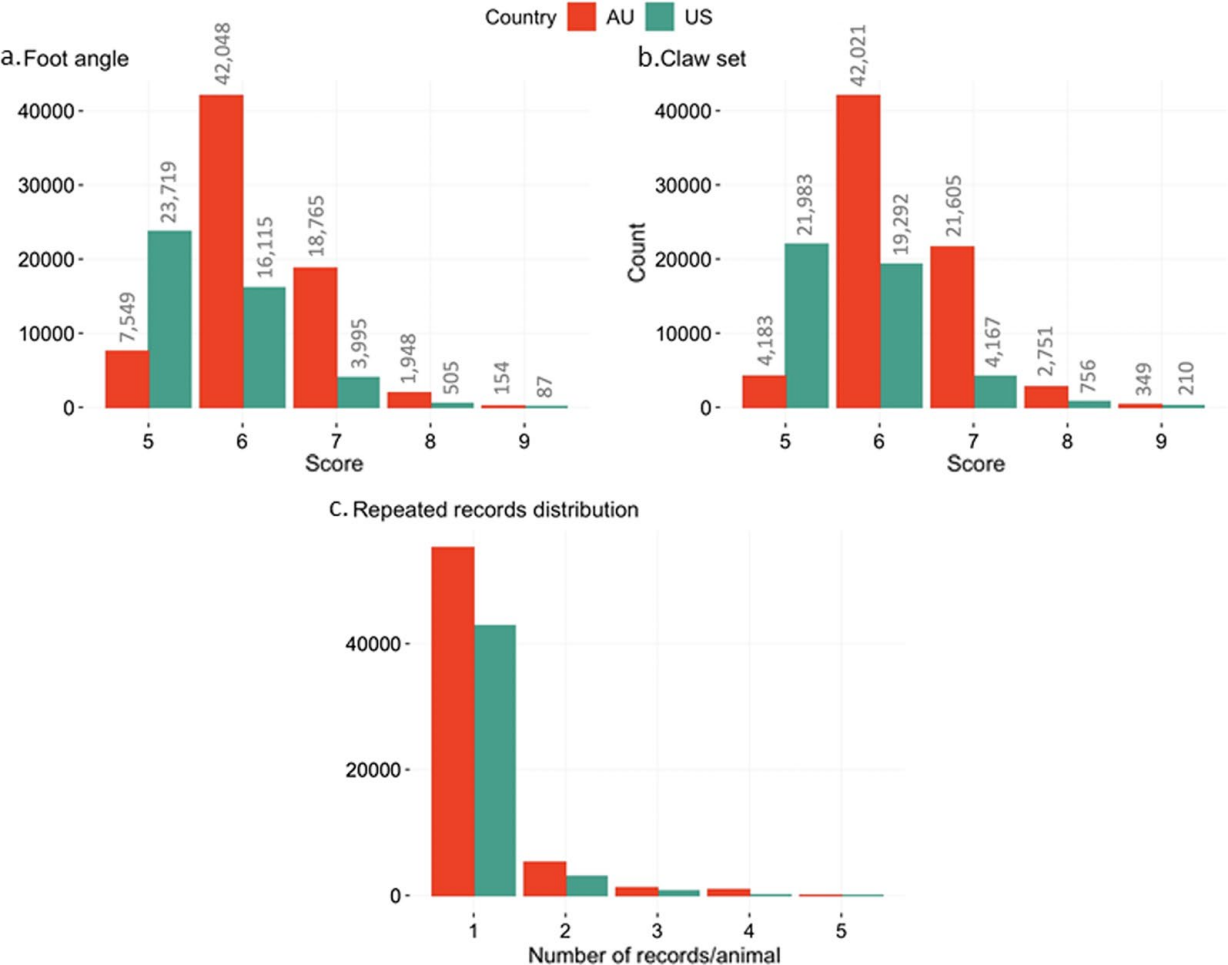
Distribution of foot scores. **a** Foot angle; **b** claw set in Angus cattle per country (AU = Australia and US = United States) based on the number of records per animal (**c**)

The phenotypic datasets included data from purebred Angus cattle registered in the AAA (referred to as the US Angus population) and AAU (referred to as the AU Angus population). In total, 85,549 records (75,020 animals; recorded from April 2014 to July 2021) from the US population and 85,439 records (74,240 animals; recorded from March 1996 to August 2021) from the AU population were available for the analyses. The description of the phenotypic datasets prior to quality control is in Additional file 1: Table S1. The datasets were edited to maintain enough variation within each level of CG (i.e., 20 records and at least two-foot score categories). Furthermore, phenotypes recorded on animals younger than 320 and older than 5475 days were removed from the analyses. Only scores from 5 to 9 were evaluated in this study because there were 52 records for FA and 383 records for CS for scores from 1 to 4 in the raw AU Angus dataset (see Additional file 1: Table S1). Furthermore, other studies have observed a decrease in the heritability estimates when scores from 1 to 4 were included and, consequently, previous researchers have applied similar filtering criteria [25, 26]. After the quality control, 44,421 FA and 46,408 CS US Angus records (from November 2014 to June 2021) and 70,464 FA and 70,909 CS AU Angus records (recorded from March 1996 to August 2021) remained for further analyses. The average (±SD) number of records per animal was 1.1 (0.30) and 1.2 (0.53), and 3814 and 7409 animals had more than one measurement for US and AU, respectively. Figure 2a, b show the distribution of FA and CS scores, respectively. Figure 2c shows the distribution of repeated records and Table 1 presents an additional description of the dataset after data quality control. The distribution of the animals’ age at recording is presented in Additional file 2: Fig. S1.

**Table 1:** Descriptive statistics of the phenotypic datasets (after quality control) of foot scores in American and Australian Angus cattle

	Foot angle		Claw set	
	US	AU	US	AU
Number of records	44,421	70,464	46,408	70,909
Number of animals	40,466	60,158	42,621	60,561
Age (days)				
$\bar{x}$ (SD)	761 (678)	701 (432)	716 (624)	699 (430)
Min	320	320	320	320
Max	4987	4821	4957	4821
Reference population	29,444	21,434	31,799	21,612

*US* American Angus registered animals, *AU* Australian Angus registered animals, *reference population* number of animals with both genomic and phenotypic information

#### Genomic datasets

The genomic dataset contained animals from multiple countries and various genotyping platforms or SNP panel densities. The genotypes were mapped to the bovine genome assembly UMD3.1. Genotype imputation was performed to a common 50K SNP panel, as implemented in the analytical pipeline of the AAA. Genotype imputation and quality control were done within each country. A quality control of the genomic data was performed at the SNP and animal levels, in which SNPs with a minor allele frequency higher than 0.05 and a call rate higher than 0.90 were kept for further analyses. Animals with a sample call rate higher than 0.80 were also kept. In total, 39,595 SNPs remained after quality control, and 1,145,751 genotyped animals remained for the genomic prediction analyses, containing 996,329 US, 131,951 AU, 17,403 Canadian, and 68 from other countries. Finally, the genomic coordinates were converted to the ARS-UCD1.2 bovine genome assembly [27, 28] using the *biomaRt* R package [29].

Only a subset of the genotyped population was used to obtain p-values for SNPs in the ssGWAS analyses because p-values depend on the prediction error variance of the SNP effect from the ssGBLUP results, and the full coefficient matrix needs to be inverted [30]. To overcome this limitation, 12,500 genotyped animals with phenotypic records from each country (25,000 genotyped animals in total) were randomly sampled.

#### Pedigree datasets

The pedigree of US and AU were merged based on a common identification number across countries, which was provided by both the AAA and AAU breeders’ associations. For the estimation of genetic parameters and ssGWAS, 122,471 and 119,160 animals for US and AU, respectively, were included in the pedigree. The pedigree file included up to four generations of ancestors from the animals with phenotypic information, including 10,945 sires in the US and 8885 in the AU pedigree dataset. In total, 973 sires had progeny in both US and AU datasets, accounting for 41% (50,205 animals) and 25% (30,220 animals) of animals in the US and AU pedigree files, respectively. Furthermore, 1383 dams with progeny shared between both countries were available, accounting for 4115 US and 2069 AU animals in the pedigree. Regarding the phenotypic data, 215 sires with progeny with phenotypic information in both countries were available, accounting for 27% of the animals with phenotypic records in the US dataset (14,121 phenotypes, which is equivalent to 12,798 animals) and 13% of animals with phenotypic records in the AU dataset (8792 phenotypes, equivalent to 7978 animals). For the genomic prediction analyses, the pedigree file was created by including up to four generations of ancestors from phenotyped animals (with and without genomic information) and relatives of genotyped animals without phenotypes, resulting in a total of 1,902,478 animals.

### Population characterization and admixture analyses

#### Linkage disequilibrium and consistency of gametic phase

The level of linkage disequilibrium (LD) was measured based on the squared correlation (r^2^) between two alleles at different loci [31], and the LD analyses were done within country. The LD decay was represented by the average of the SNP-pairwise LD sorted into SNP bins of 100 kbp. All genotyped animals for both countries were included in the analyses (1.12 million animals). The consistency of gametic phase was calculated as the Pearson correlation coefficient between the average of a signed LD by bins of US and AU populations. The signed-LD was calculated as the square root of the r^2^ values with the sign of the disequilibrium metric $[D=f(AB)-f(A)f(B)]$, considering two loci on a chromosome with alleles *A*, *a* and *B*, *b*; $f(A)$ is the allele frequency of *A* at one locus, $f(B)$ is the allele frequency of *B* at a different locus, and $f(AB)$ is the frequency of the *AB* haplotype. Both metrics were calculated using the snp1101 software [32].

#### Principal components analysis

Principal component (PC) analysis (PCA) was performed to investigate the genomic similarities between the US and AU Angus populations. The PC were estimated based on the genomic relationship matrix ($\mathbf{G}$, calculated as in the first method proposed by VanRaden [33]) and using the snp1101 software [32]. The software snp1101 implements an approach called fast PCA, which is less memory-intensive given a large-scale dataset compared to the traditional PCA. The fast PCA randomizes the top N PC, and for this study, we considered the top 10 PC. The subset of animals used to create the $\mathbf{G}$ matrix and to obtain the eigen value decomposition for the PCA included: (i) all genotyped animals from the US and AU datasets (~ 1.2 million animals); (ii) only the animals with phenotypic and genomic information (Tables 1 and 59K animals); (iii) 100,000 animals randomly sampled from each US and AU dataset; and (iv) 10,000 animals randomly sampled from each US and AU dataset. The scenarios (iii) and (iv) were performed as supplementary analyses to verify the pattern in the PCA when a balanced number of genotyped individuals from both countries and a random selection from the large genomic dataset were used.

#### Admixture analyses

The population structure for each country was assessed using the ADMIXTURE software [34, 35]. The total number of genotyped animals included in these analyses was 20,000 with 50% from the US and 50% from the AU datasets. LD pruning was performed, i.e. SNPs from pairs with r^2^ higher than 0.20 in a genomic window of 10 SNPs were filtered out. In total, 13,566 SNPs remained for the admixture analyses. The optimal K value was defined based on a fivefold cross-validation procedure and prior knowledge about the populations, in which the K values evaluated ranged from 1 to 10 (see Additional file 2: Fig. S2). The K value with the lowest slope-decay of cross-validation (CV) error was assumed as the optimal number of ADMIXTURE clusters (K = 3).

#### Estimates of genetic parameters between countries

In total, three main groups of genetic parameter analyses were performed. First, we evaluated a within-country two-trait model (WC), in which the datasets from each country were analyzed separately (e.g., US or AU populations) and a covariance structure was considered between FA and CS [Eq. (1) description (a)]. The AAA currently runs a two-trait (FA and CS) model for genetically evaluating foot scores. Equation (1) is:

1 $${\mathbf{y}}={\mathbf{X}}{\mathbf{\upbeta }}+\mathbf{Za}+{\mathbf{Wpe}}+{\mathbf{e}}.$$

Description (a) of Eq. (1) for a within country two-trait (WC-TT) model: $\mathbf{y}$ is a two-trait vector with phenotypic records for FA and CS, $\varvec{\upbeta}$ is a vector of fixed effects for both traits, including age (in days) as a linear covariate and contemporary group, $\mathbf{a}$ is a vector of the animal random effect with $\mathbf{a} \sim N(\mathbf{0},\mathbf{\Sigma } \otimes \mathbf{A})$, $\mathbf{pe}$ is a vector of the random permanent environmental effect with $\mathbf{pe} \sim N(\mathbf{0}, \mathbf{Q}\otimes \mathbf{I})$, and $\mathbf{e}$ is a vector of the random residual with $\mathbf{e} \sim N(\mathbf{0}, \mathbf{R}\otimes \mathbf{I})$. $\mathbf{X}$, $\mathbf{Z}$, and $\mathbf{W}$ are the incidence matrices for $\varvec{\upbeta }$, $\mathbf{a}$, and $\mathbf{pe}$, respectively. ⊗ is the Kronecker product, $\mathbf{A}$ is a pedigree-based relationship matrix, $\mathbf{I}$ is an identity matrix, $\mathbf{\Sigma }$ is a covariance matrix for $\mathbf{a}$ as described in Eq. (2), $\mathbf{R}$ is the residual covariance matrix as in Eq. (3), and $\mathbf{Q}$ is the permanent environment covariance matrix as in Eq. (4).

2 $$\varvec{\Sigma}=\left[\begin{array}{cc}{\sigma }_{{\text{a}}_{\text{FA}}}^{2}& {\sigma }_{\text{a}}\\ {\sigma }_{\text{a}}& {\sigma }_{{\text{a}}_{\text{CS}}}^{2}\end{array}\right],$$

3 $$\mathbf{R}=\left[\begin{array}{cc}{\sigma }_{{\text{e}}_{\text{FA}}}^{2}& {\sigma }_{\text{e}}\\ {\sigma }_{\text{e}}& {\sigma }_{{\text{e}}_{\text{CS}}}^{2}\end{array}\right],$$

4 $$\mathbf{Q}=\left[\begin{array}{cc}{\sigma }_{{\text{p}\text{e}}_{\text{FA}}}^{2}& {\sigma }_{\text{p}\text{e}}\\ {\sigma }_{\text{p}\text{e}}& {\sigma }_{{\text{p}\text{e}}_{\text{CS}}}^{2}\end{array}\right],$$

where $\text{a}$, $\text{p}\text{e}$ and $\text{e}$ correspond to the additive genetic, permanent environment, and residual components, respectively.

Second, a multi-country two-trait model (MC-TT) was analyzed [Eq. (1) description (b)], in which data from US and AU were analyzed jointly with a covariance structure of FA and CS. Description (b) of Eq. (1) for a MC-TT model: $\mathbf{y}$ is a four-trait vector (i.e., concatenation of two countries and two foot score traits), $\varvec{\upbeta }$ is a vector of fixed effects, including age (in days) as a linear covariate and contemporary group. $\varvec{\Sigma }$ is a covariance matrix for $\mathbf{a}$ as described in Eq. (5), $\mathbf{R}$ is the residual covariance matrix as in Eq. (6), and $\mathbf{Q}$ is the permanent environment covariance matrix as in Eq. (7). The other terms are as defined above.

5 $$\varvec{\Sigma}=\left[\begin{array}{*{20}{c}}{{\sigma}_{{\text{a}}_{{\text{FA}}_{\text{US}}}}^{2}}& {{\sigma}_{{\text{aFA}}_{\text{US}}{{\text{CS}}_{\text{US}}}}}& {{\sigma}_{{\text{aFA}}_{\text{US}}{{\text{FA}}_{\text{AU}}}}}& {{\sigma}_{{\text{aFA}}_{\text{AU}}{{\text{CS}}_{\text{AU}}}}}\\ {{\sigma}_{{\text{aFA}}_{\text{US}}{{\text{CS}}_{\text{US}}}}}& {{\sigma}_{{\text{a}}_{{\text{CS}}_{\text{US}}}}^{2}}& {{\sigma }_{{\text{aCS}}_{\text{US}}{{\text{FA}}_{\text{AU}}}}}& {{\sigma}_{{\text{aCS}}_{\text{US}}{{\text{CS}}_{\text{AU}}}}}\\ {{\sigma}_{{\text{aFA}}_{\text{US}}{{\text{FA}}_{\text{AU}}}}}& {{\sigma}_{{\text{aCS}}_{\text{US}}{{\text{FA}}_{\text{AU}}}}}& {{\sigma}_{{\text{a}}_{\text{FA}_{\text{AU}}}}^{2}} & {{\sigma}_{{\text{aFA}}_{\text{AU}}{{\text{CS}}_{\text{AU}}}}} \\ {{\sigma}_{{\text{aFA}}_{\text{AU}}{{\text{CS}}_{\text{AU}}}}}& {{\sigma}_{{\text{aCS}}_{\text{US}}{{\text{CS}}_{\text{AU}}}}} & {{\sigma}_{{\text{aFA}}_{\text{AU}}{{\text{CS}}_{\text{AU}}}}} & {{\sigma}_{{\text{a}}_{{\text{CS}}_{\text{AU}}}}^{2}}\end{array}\right],$$

6 $${\mathbf{R}} = \left| {\begin{array}{*{20}c} {\sigma_{{{\text{e}}_{{\text{FA}_{\text{US}} }} }}^{2} } & {\sigma_{{{\text{eFA}}_{{{\text{US}}}} {\text{CS}}_{{{\text{US}}}} }} } & 0 & 0 \\ {\sigma_{{{\text{eFA}}_{{{\text{US}}}} {\text{CS}}_{{{\text{US}}}} }} } & {\sigma_{{{\text{e }}_{{\text{CS}_{\text{US}} }} }}^{2} } & 0 & 0 \\ 0 & 0 & {\sigma_{{{\text{e}}_{{\text{FA}_{\text{AU}} }} }}^{2} } & {\sigma_{{{\text{eFA}}_{{{\text{AU}}}} {\text{CS}}_{{{\text{AU}}}} }} } \\ 0 & 0 & {\sigma_{{{\text{eFA}}_{{{\text{AU}}}} {\text{CS}}_{{{\text{AU}}}} }} } & {\sigma_{{{\text{e}}_{{\text{CS}_{\text{AU}} }} }}^{2} } \\ \end{array} } \right|,$$

7 $${\mathbf{Q}} = \left| {\begin{array}{*{20}c} {\sigma_{{{\text{pe}}_{{\text{FA}_{\text{US}} }} }}^{2} } & {\sigma_{{{\text{peFA}}_{{{\text{US}}}} {\text{CS}}_{{{\text{US}}}} }} } & 0 & 0 \\ {\sigma_{{{\text{peFA}}_{{{\text{US}}}} {\text{CS}}_{{{\text{US}}}} }} } & {\sigma_{{{\text{pe }}_{{\text{CS}_{\text{US}} }} }}^{2} } & 0 & 0 \\ 0 & 0 & {\sigma_{{{\text{pe }}_{{\text{FA}_{\text{AU}} }} }}^{2} } & {\sigma_{{{\text{peFA}}_{{{\text{AU}}}} {\text{CS}}_{{{\text{AU}}}} }} } \\ 0 & 0 & {\sigma_{{{\text{peFA}}_{{{\text{AU}}}} {\text{CS}}_{{{\text{AU}}}} }} } & {\sigma_{{{\text{pe }}_{{\text{CS}_{\text{AU}} }} }}^{2} } \\ \end{array} } \right|,$$

Third, a joint-country two-trait model was analyzed (JC-TT), in which the data for both FA and CS from the US and AU populations were combined as if they were a single population [Eq. (1) Description (c)]. Description (c) of Eq. (1) for JC-TT: $\mathbf{y}$ is a two-trait vector (e.g., FA and CS), $\varvec{\upbeta}$ is a vector of fixed effects for both traits, including age (in days) as a linear covariate nested within country and CG (no CG overlapped between US and AU). $\mathbf{\Sigma }$ is a covariance matrix for $\mathbf{a}$ as described in Eq. (2), $\mathbf{R}$ is the residual covariance matrix as in Eq. (3), and $\mathbf{Q}$ is the permanent environment covariance matrix as in Eq. (4). Age was nested within country due to differences in the age at recording between US and AU (see Additional file 2: Fig. S1), which could be a consequence of differences in the production systems. For the estimation of the (co)variance components, the airemlf90 (AIREML analyses using the default settings) package from the blupf90+ program family was used [36, 37]. Finally, within-country single-trait (WC-ST), multi-country single-trait (MC-ST), and joint-country single-trait (JC-ST) models were also fitted, where a null covariance between FA or CS was considered.

### Genomic prediction of breeding values

Identifying the optimal scenarios for performing genomic prediction of breeding values for FA and CS in the US and AU Angus populations was a key goal of this study. The datasets used mimic the current genetic evaluation performed by AAA. A single-step GBLUP (ssGBLUP) approach was used for genomic predictions. A forward genomic evaluation was performed, in which animals born in 2020 for the US and 2019–2020 for AU were considered as the target (or validation) population. A simplification of the linear regression method [38] was used to assess the predictive ability of the scenarios evaluated based on bias (Eq. 8), dispersion (Eq. 9), and accuracy (Eq. 10) of the GEBV. In the original method [38], the denominator of Eq. (10) is $\left(1+\bar{F}-2f\right){V}_{g}$, in which $2f$ is the average relationship between individuals and ${V}_{g}$ is the additive genetic variance obtained based on the partial dataset. First, a common and unchangeable genomic dataset was considered across all genomic prediction scenarios evaluated. Inversion of the genomic relationship matrix $(\mathbf{G})$ with 1,145,751 genotyped animals would not be feasible. Therefore, the algorithm of proven and young animals (APY; [39]) was used and 22,000 randomly sampled animals out of the whole pool of genotyped animals available were attributed to the core group. The core animals were the same across all scenarios.

8 $$bias= \overline{{GEB{V}_{partial}}}-\overline{{GEB{V}_{whole}}},$$

9 $$dispersion=\frac{cov(GEB{V}_{partial},GEB{V}_{whole})}{var\left(GEB{V}_{partial}\right)},$$

10 $$acc=\sqrt{\frac{cov(GEB{V}_{partial},GEB{V}_{whole})}{\left(1-\bar{F}\right){\sigma }_{u}^{2}}},$$

where $\overline{{GEB{V}_{partial}}}$ is the average of the GEBV of validation animals using the partial dataset (i.e., with the phenotypes from the validation group masked), $\overline{{GEB{V}_{whole}}}$ is the average of the GEBV of validation animals using the whole dataset (i.e., the phenotypes from the validation group were also included in the analyses), $\bar{F}$ is the average inbreeding of the validation group, and ${\sigma }_{u}^{2}$ is the additive genetic variance calculated using the whole dataset.

The genomic prediction scenarios changed depending on the phenotypic datasets included in the training population, statistical models, and estimates of genetic parameters. The scenarios tested are described in Table 2. In brief, the main groups of analyses were: (1) within-country genomic evaluations (WC) based on single-trait (WC-ST) or two-trait (WC-TT) models, in which the analyses are done separately for each country; (2) multi-country genomic evaluations (MC) based on single-trait (MC-ST) or two-trait (MC-TT) models, in which the analyses are done combining the datasets from both countries and considering them as potentially correlated traits (covariance structure among traits); (3) joint-country genomic evaluations (JC), based on single-trait (JC-ST) or two-trait (JC-TT) models, in which the analyses are done combining the phenotypic records from both countries for each trait as if AU and US were a single Angus population; and, (4) across-country genomic evaluations (AC), based on two-trait (AC) models, in which the records from one country are considered in the training population to predict the GEBV of animals from a different country.

For these analyses, the blup90iod2OMP1 package [36, 37] was used to obtain the GEBV from the whole and partial datasets as described by Legarra and Reverter [38]. During the genomic prediction analyses using the blup90iod2OMP1 package, the following flags were used: (1) no direct inversion of the pedigree relationship matrix containing the genotyped animals (i.e., ${ \mathbf{A}}_{\mathbf{22}}$); (2) $\mathbf{G}$ (genomic relationship matrix) was blended with ${ \mathbf{A}}_{\mathbf{22}}$ as $0.90 \mathbf{G}+0.10{ \mathbf{A}}_{\mathbf{22}}$ before matrix inversion (i.e., alpha equal to 0.90 and beta equal to 0.10); (3) use of a sparse matrix package (YAMS) for approximation of the inversion of the left hand side of the mixed model equations; and (4) the threshold for inversion was the default value (10^−11^). The approximated theoretical (or individual) accuracies (by inversion of the coefficient matrix of the mixed model equations) were obtained from the accf90GS package [36, 37], and the approximation was based on the diagonal elements in the left-hand-side of the mixed model equations after absorbing the non-genetic effects (methodology described in detail by Misztal and Wiggans [40]).

In the AC scenario (AC), data from Canadian Angus (part of the AAA database) were included for validation purposes. The whole dataset analyses based on the Legarra and Reverter [38] approach was performed for the scenarios including phenotypic and genotypic information from all three countries (full dataset; best approximation of the true breeding values), followed by a partial analysis containing only the phenotypic records from the estimation set country (either US or AU).

**Table 2:** Description of the genomic prediction scenarios evaluated for foot angle (FA) and claw set (CS) in Australian (AU) and United States (US) Angus cattle

Abbreviation	Description	Scenarios	N_TOT_	Target
WC-TT	Within-country two-trait model	FA_US.WC_	44,421	2020^a^:4142
	Single-country analysis (no correlation between US and AU traits) for FA and CS (FA and CS are correlated traits)	CS_US.WC_	46,408	2020:4429
		FA_AU.WC_	70,464	2020:325
				2019:3678
		CS_AU.WC_	70,909	2020:339
				2019:3675
MC-TT	Multi-country two-trait model	FA_US.MC_	44,421	2020^3^:4142
	Two-country and two traits (FA and CS from US and AU are correlated) model	CS_US.MC_	46,408	2020:4429
		FA_AU.MC_	70,464	2020:325
				2019:3678
		CS_AU.MC_	70,909	2020:339
				2019:3675
JC-TT	Joint countries two-trait model	FA_JC_	114,885	2020:4467
	Single-trait analysis in which the phenotypic records for each trait (FA and CS) from both countries were combined as if the US and AU were the same population (FA and CS are correlated)			2019:3678
		CS_JC_	117,317	2020:4768
				2019:3675
AC	Across-country two-trait model	FA_AC-EsetUS.TargetAU_	44,421	2020:325
	Across-country analysis for FA and CS (FA and CS are correlated traits). In this case, two scenarios:			2019:3678
	(1) Genomic model predicting AU and Canadian Angus (CA) (AU and CA are the prediction target—Target) based on genomic and phenotypic information from US animals (US is the Training estimation set—Eset)	FA_AC-EsetUS.TargetCA_	44,421	700
	(2) Genomic model predicting US and CA (US and CA are the Target) based on a genomic and phenotypic information from AU animals (AU is the Eset)	FA_AC-EsetAU.TargetUS_	70,464	2020:4142
		FA_AC-EsetAU.TargetCA_	70,464	700
		CS_AC-EsetUS.TargetAU_	46,408	2020:339
				2019:3675
		CS_AC-EsetUS.TargetCA_	46,408	700
		CS_AC-EsetAU.TargetUS_	70,909	2020:4429
		CS_AC-EsetAU.TargetCA_	70,909	700

For the target population numbers presented in this table, all the target animals had both phenotypic and genotypic information. Furthermore, for the purpose of the forward validation, the remaining animals born in 2020 for US or in 2019–20 for AU with phenotype and no genotype were removed from the partial-dataset analyses to avoid biasing the results

N_TOT_ : number of animals included in the whole-dataset analyses; Target: animals in the target population, in which their phenotypic records were masked in the partial-dataset analyses

^a^Year of birth of the target animals

### Single-step genome-wide association studies

Single-step genome-wide association studies (ssGWAS) were performed based on multi-country single-trait models (MC-ST). First, pedigree-based genetic parameters were obtained for the MC-ST model, and then the GEBV were estimated using those parameters. Subsequently in the ssGWAS step, SNP effects and p-values were calculated using the postGSf90 package from the blupf90+ family of programs [30, 37, 41, 42]. All animals with phenotypic records were included in the analyses (see Table 1) and the pedigree traced back up to four generations from the animals with phenotypic information. A Bonferroni correction was used to account for multiple testing, in which two thresholds were defined to determine SNP significance, including 10^−6^ and 10^−4^ for strong and moderate associations, respectively.

A genomic window of 100 kb on both sides of the significant SNPs was considered for identifying associated genes and performing functional analyses. Gene symbols were retrieved from Ensembl using the *biomaRt* R package [29, 43]. Functional annotation was performed in terms of gene ontology (GO) biological processes (GO_BP; [44]) and metabolic pathways of the Kyoto encyclopedia of genes and genomes (KEGG; [45]) available in the DAVID database (david.ncifcrf.gov/tools.jsp [46]; accessed 16 August 2022). Genome coordinates were based on the ARS-UCD1.2 bovine genome assembly [27, 28].

## Results

### Population characterization, clustering, and admixture analyses

**Fig. 3:**
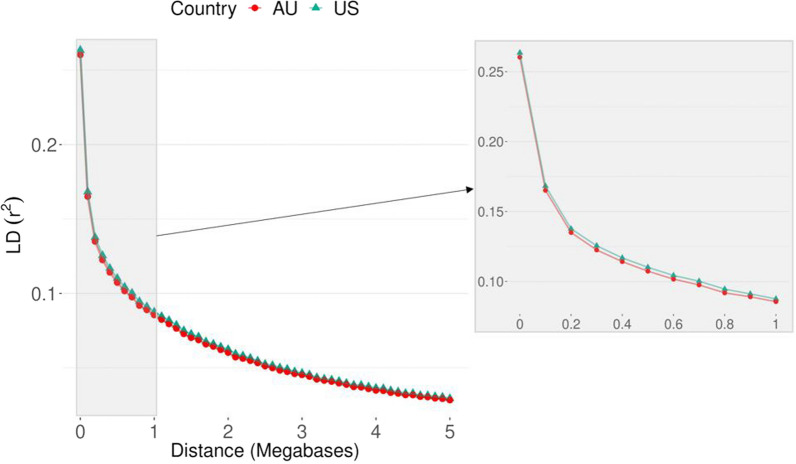
Linkage disequilibrium decay for the United States (US) and Australian (AU) Angus populations considering all genotyped animals available from both countries (~ 1.2 million genotyped animals)

The LD decay for the US and AU Angus populations is shown in Fig. 3, and in general, it had a similar pattern in both populations. The average LD (±SD) at an average distance between adjacent SNPs of 65 kb in the SNP panel used was 0.23 (0.27) and 0.23 (0.26) for the US and AU Angus populations, respectively.

**Fig. 4:**
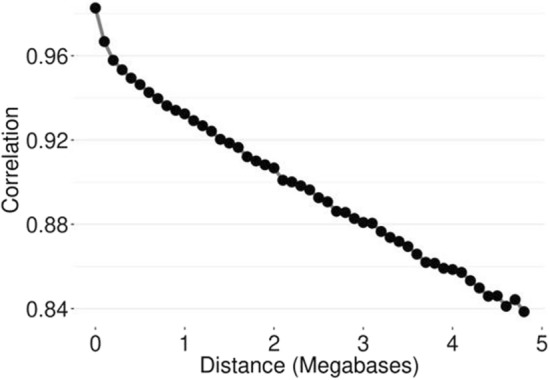
Consistency of gametic phase between US and AU Angus populations

Figure 4 presents the consistency of gametic phase between both populations, which is a correlation metric accounting for the magnitude of the LD and the similarity of linkage phase between genomic markers across populations. Higher values indicate that the SNP effects are likely to be more similar across the populations. Based on the results obtained (Fig. 4), the US and AU Angus populations seem to share similar gametic phase patterns. Even markers separated by 5 Mb have a consistency of gametic phase greater than 0.80. In general, the first 10 PC were not sufficient to separate (if there was any stratification) the US and AU populations (data not shown). The top 10 PC together explained a maximum of 6% of the total variation of $\mathbf{G}$ (see Additional file 1: Table S2).

**Fig. 5:**
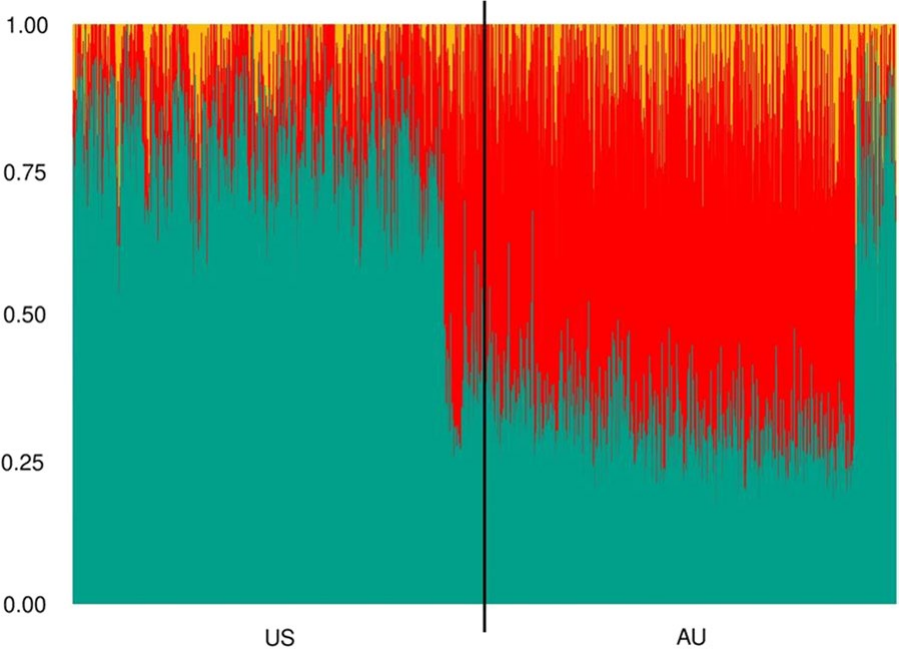
Admixture analysis for US and AU Angus populations (K = 3)

Figure 5 shows the admixture analysis for the US and AU Angus cattle populations. Three common genetic groups were identified for both populations. For the US animals, one genetic group (green color; Fig. 5) largely contributed to the recent US population compared to a small contribution from the other two populations (red and yellow colors). Two genetic groups (green and red colors; Fig. 5) explained similar amount of variation of the AU recent population, and a small contribution coming from the third genetic group (yellow color; Fig. 5). For completeness, Additional file 2: Fig. S3 presents the plots of admixture analyses considering two (K = 2) and six (K = 6) ancestral population clusters.

### Estimates of genetic parameters

#### Within-country models

Table 3 presents the estimates of heritability, repeatability, and genetic correlation between FA and CS using the WC-TT model. Moderate heritability and repeatability estimates were observed for both foot score traits ranging from 0.22 to 0.26 and from 0.30 to 0.35 (Table 3), respectively. In each country, favorable, positive, and moderate genetic correlations were observed between FA and CS (i.e., 0.50 for US and 0.46 for AU; Table 3). The genetic parameters for FA and CS obtained with the WC-TT or WC-ST models were similar (Table 3 and see Additional file 1: Table S3).

**Table 3:** Estimates of heritability $({\text{h}}^{2})$, repeatability $(\text{rep})$, and genetic correlation $({\text{r}}_{\text{g}})$ between foot angle (FA) and claw set (CS) scores in the Australian (AU) and American (US) Angus populations based on within-country two-trait (WC-TT) models

Trait	${\text{h}}^{2}$	$\text{rep}$	${\text{r}}_{\text{g}}$
FA_US_	0.22 (0.01)^a^	0.32 (0.01)	0.50 (0.04)
CS_US_	0.21 (0.01)	0.30 (0.01)	
FA_AU_	0.24 (0.01)	0.30 (0.01)	0.46 (0.03)
CS_AU_	0.26 (0.01)	0.35 (0.01)	

^a^Values within parentheses represent the standard error estimates

#### Multi-country models

The genetic parameters for the MC analyses are in Additional file 1: Table S4. The heritability estimates for FA and CS were similar to those reported in Table 3 (WC-TT model). The genetic correlation between the US and AU traits was 0.61 (0.10) for FA and 0.76 (0.07) for CS (see Additional file 1: Table S4). Adding genomic information into the MC-ST model did result in a reduction in the heritability estimates for the US traits (from 0.22 to 0.18 for FA and from 0.22 to 0.18 to CS) and for CS from AU (from 0.26 to 0.25; Additional file 1: Table S5). However, the genomic-based genetic correlations between the US and AU traits increased for both FA (0.76) and CS (0.78) (see Additional file 1: Table S5) compared to the MC-TT analyses (see Additional file 1: Table S4).

#### Joint countries models

The heritability estimates of FA and CS when considering the JC-ST model were 0.24 (0.01) and 0.25 (0.01), respectively. The heritability estimates for the JC-TT analyses were 0.24 for both FA and CS and the genetic correlation between both traits was 0.46 (see Additional file 1: Table S6), which were similar to the estimates obtained using the WC (Table 3) and MC (see Additional file 1: Table S4) models. Furthermore, similar heritability estimates were obtained for FA and CS when both traits were analyzed separately (see Additional file 1: Table S4).

### Genomic prediction of breeding values

**Fig. 6:**
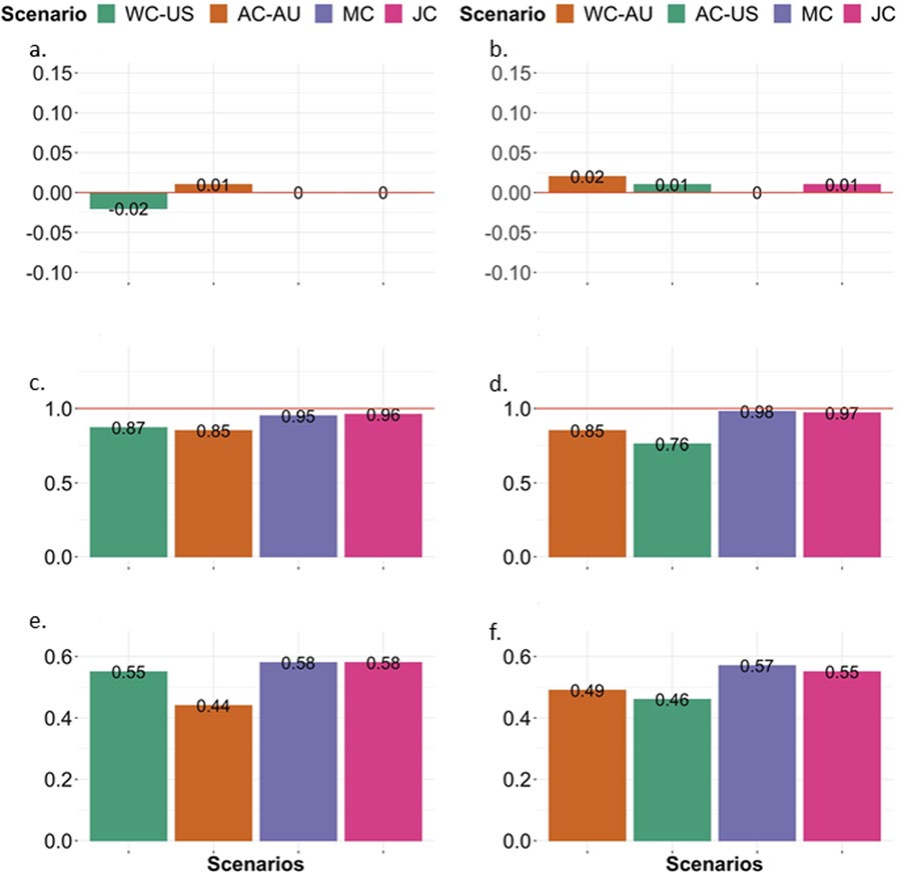
Foot score: predictive ability of a two-trait model of foot angle (FA) and claw set (CS) considering the target population as the US (**a**, **c**, and **e**) and AU (**b**, **d**, and **f**) Angus cattle populations. “US target” represents American Angus target animals (born in 2020), and “AU target” are Australian Angus target animals born in 2019 and 2020. WC-US within country two-trait model using US phenotypic data, AC-AU across country two-trait model using AU to predict US, MC multi-country two-trait model, JC joint-country two-trait, WC-AU within country two-trait model using AU phenotypic data, AC-US across country two-trait model using US to predict AU

**Fig. 7:**
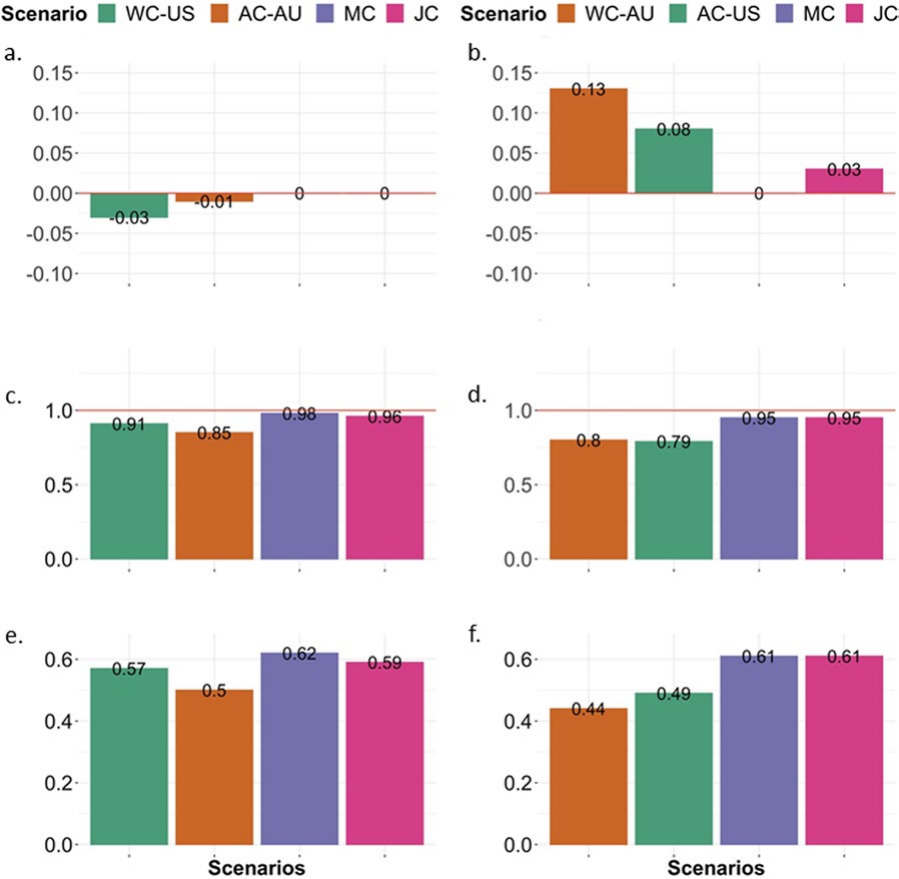
Claw set: predictive ability of a two-trait model of foot angle (FA) and claw set (CS) considering the target population as the US (**a**, **c**, and **e**), and as AU (**b**, **d**, and **f**) Angus cattle populations. “US target” represents American Angus target animals (born in 2020), and “AU target” are Australian Angus target animals born in 2019 and 2020. WC-US within country two-trait model using US phenotypic data, AC-AU across country two-trait model using AU to predict US, MC multi-country two-trait model, JC joint-country two-trait, WC-AU within country two-trait model using AU phenotypic data, AC-US across country two-trait model using US to predict AU

For both FA and CS, joint US and AU genomic predictions (either MC or JC analyses) yielded higher accuracies than WC (Figs. 6 and 7 for FA and CS, respectively, and Additional file 1: Tables S7–S10). The AC for Canadian Angus is presented in Additional file 1: Tables S7–S10. The US estimation set resulted in a higher prediction accuracy for Canadian Angus (FA: 0.80 and CS: 0.85) than the AU estimation set (FA: 0.77 and CS: 0.76), which was expected due to the greater connectedness between US and Canadian Angus populations. The MC-TT and JC-TT models resulted in similar accuracies for AU or US as validation sets (Additional file 1: Tables S9 and S10).

Similar patterns were observed when MC, JC, and WC were analyzed in the context of single-trait analyses (FA or CS, see Additional file 1: Tables S11 and S12). Furthermore, on average, a joint US and AU analysis also resulted in greater individual GEBV accuracy (theoretical accuracy) compared to WC evaluations (see Additional file 1: Tables S13 and S14).

The Pearson and Spearman correlations of proven sires’ GEBV (sires with more than 50 progeny with records that were raised in either one of the countries or in both of them) were calculated, with high correlations (> 0.75) being observed across all scenarios (see Additional file 1: Tables S15 and S16). The Spearman correlations ranged from 0.94 to 0.99 between a model accounting for GxE (MC analyses) and a model considering both US and AU as a single population (JC analyses). These results indicate that there is lower re-ranking of top breeding animals when the data from both countries are combined either through MC or JC analyses.

### Single-step genome-wide association studies

**Fig. 8:**
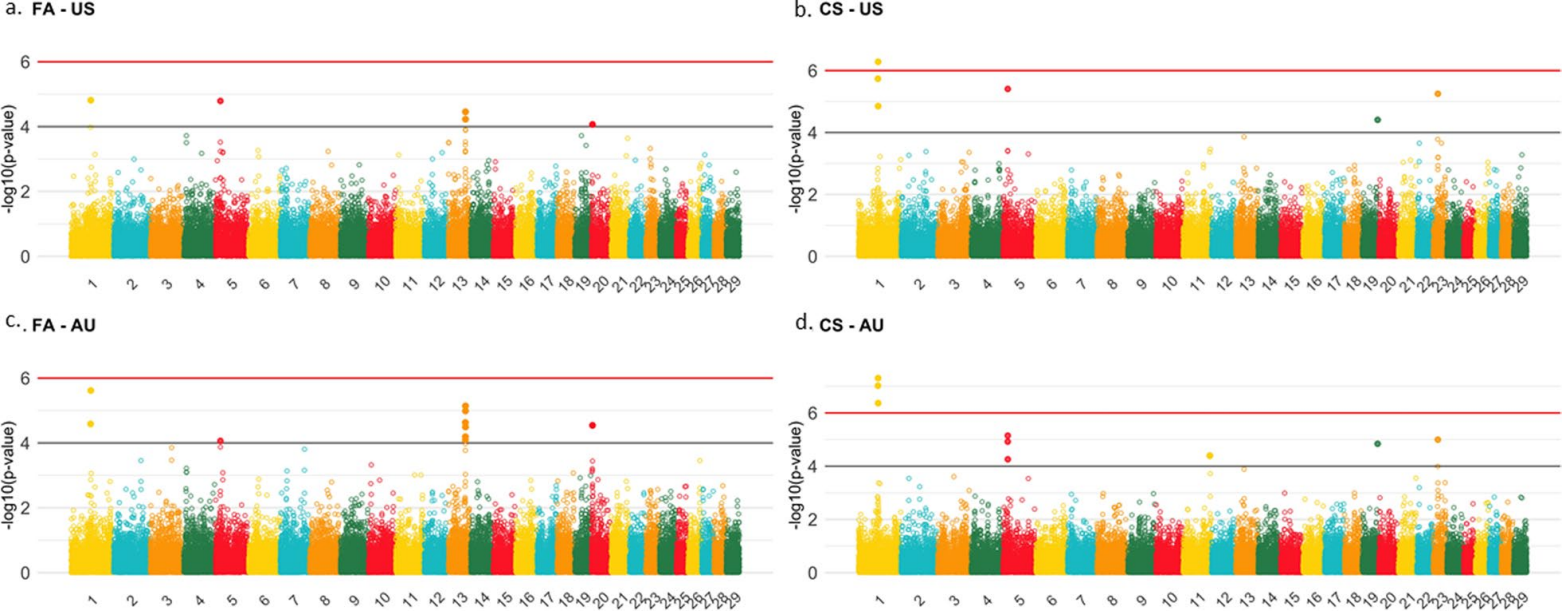
Manhattan plot for foot angle and claw set considering a two-trait model for US and AU Angus cattle populations

Figure 8 presents the Manhattan plots of FA and CS for both US and AU populations based on the MC-ST models. Therefore, SNP effects and p-values were obtained for each trait-population. Five and 12 SNPs were significantly associated with FA in US and AU populations, respectively (see Additional file 1: Table S17). For both the US and AU populations, the SNPs were located on *Bos taurus* (BTA) chromosomes BTA1, 5, 13, and 20 (see Additional file 1: Table S17). Sixteen and 35 genes were annotated within the regions spanning 100 kb up- and downstream of the position of the significant SNPs (Table 4 and Additional file 1: Table S17). Among the genes annotated for FA for the US and AU populations, none were enriched in GO terms and pathways. The GO biological terms and pathways in which those genes are involved are listed in Additional file 1: Table S18.

Six and nine SNPs were significantly associated with CS in the US and AU populations (see Additional file 1: Table S19), respectively. The SNPs were located on BTA1, 5, 19, and 23 for both the US and AU populations, and for the AU populations SNPs on BTA11 were also found (Fig. 8 and see Additional file 1: Table S19). There were seven and 14 genes annotated for those SNPs ($\pm$ 100 kb) for the US and AU populations (Table 5), respectively. None of the genes annotated for CS were enriched in GO terms and pathways (see Additional file 1: Table S20). The Q–Q plots for the p-values of SNPs are in Additional file 2: Fig. S4.

**Table 4:** Candidate genes identified for foot angle in the American and Australian Angus populations based on a multi-country single-trait (MC-ST) model

Chromosome	Gene symbol	Gene start:end position (bp)^a^	Population
BTA1	*ATP13A5*	74,043,235:74,159,562	AU
	*HRASLS*	74,163,666:74,183,456	AU
	ENSBTAG00000055130	74,829,775:74,829,854	US and AU
	*FGF12*	74,876,548:75,283,017	US and AU
BTA13	*NCOA6*	64,051,984:64,148,363	AU
	*GGT7*	64,158,906:64,183,221	AU
	*ACSS2*	64,186,743:64,233,568	AU
	*GSS*	64,234,188:64,259,465	AU
	*MYH7B*	64,280,737:64,309,631	AU
	*bta-mir-499*	64,292,583:64,292,680	AU
	*TRPC4AP*	64,304,949:64,374,165	AU
	*EDEM2*	64,392,791:64,420,836	AU
	*PROCR*	64,444,427:64,449,124	AU
	*MMP24*	64,512,071:64,543,097	AU
	*EIF6*	64,544,773:64,553,147	AU
	*FAM83C*	64,554,623:64,561,144	AU
	*UQCC1*	64,573,005:64,669,659	AU
	*GDF5*	64,681,475:64,685,560	US and AU
	*CEP250*	64,699,831:64,746,870	US and AU
	*C13H20orf173*	64,765,618:64,768,195	US and AU
	*ERGIC3*	64,776,869:64,791,647	US and AU
	*SPAG4*	64,841,049:64,845,754	US and AU
	*CPNE1*	64,848,971:64,884,882	US and AU
	*RBM12*	64,873,751:64,884,908	US and AU
	*NFS1*	64,887,674:64,904,921	AU
	*ROMO1*	64,904,969:64,906,500	AU
	*RBM39*	64,908,243:64,934,850	AU
BTA20	*ERGIC1*	4,596,395:4,709,455	US and AU
	*RPL26L1*	4,716,985:4,723,653	US and AU
	*ATP6V0E1*	4,732,524:4,762,537	US and AU
	*CREBRF*	4,776,513:4,831,767	US and AU

BTA: *Bos taurus* chromosome

^a^These genomic positions are based on the ARS-UCD1.2 bovine genome assembly

**Table 5:** Annotated candidate genes for claw set in the American (US) and Australian (AU) Angus populations based on a multi-country single-trait (MC-ST) model

Chromosome	Gene symbol	Start:end position (bp)^a^	Population
BTA1	*ATP13A5*	74,043,235:74,159,562	US and AU
	*HRASLS*	74,163,666:74,183,456	US and AU
	*MB21D2*	74,489,024:74,609,955	US and AU
	ENSBTAG00000053503	74,675,757:74,698,158	US and AU
	ENSBTAG00000055130	74,829,775:74,829,854	US and AU
	*FGF12*	74,876,548:75,283,017	US and AU
BTA5	*KITLG*	18,250,809:18,353,485	AU
BTA11	*ASS1*	100,770,166:100,822,252	AU
	*FUBP3*	100,882,266:100,931,850	AU
	*U6*	100,937,424:100,937,524	AU
	*PRDM12*	100,951,292:100,966,190	AU
	*EXOSC2*	100,975,458:100,985,125	AU
	*ABL1*	100,988,747:101,131,037	AU
BTA23	*SUPT3H*	18,239,634:18,641,106	US and AU

BTA: *Bos taurus* chromosome

^a^These genomic positions are based on the ARS-UCD1.2 bovine genome assembly

## Discussion

### Population structure

The success of an across-population (e.g., across-country, across-breed) genomic prediction depends on the levels of LD and genetic links between the populations [47]. The US and AU Angus populations are closely genetically related based on all the genetic analyses performed. For instance, the first 10 PC together explained less than 6% of the total variation and were not able to separate the US and AU populations into different clusters. Furthermore, 41% (50,205 animals) and 25% (30,220 animals) of the animals in the US and AU pedigree, respectively, had common sires. In addition, 27% of the US and 13% of the AU animals in the phenotypic dataset were sired from common sires across countries. Moreover, the US and AU Angus populations seem to have similar patterns of gametic phase and common ADMIXTURE genetic groups (Figs. 4 and 5).

The first record of the Angus breed in Australia was in 1824, when animals were directly imported from Scotland (www.angusaustralia.com.au/about/our-cattle/history-of-angus/; accessed 7 August 2022). Similarly, the first Angus animals were brought to the US in 1873, also directly from Scotland (www.angus.org/pub/anghist; accessed 7 August 2022). In Fig. 5, both the US and AU populations have up to three clustered genetic groups (also known as ancestral populations), in which two were predominant (in red and green). The differences in proportion of genetic grouping from ADMIXTURE between the US and AU populations could be explained by geographical and time differences of selection from the breeds, since there is a gap of 49 years between the breed introduction in the US and AU. Furthermore, the origin of the first animals introduced in the country could also be different, with slightly different genetic selection histories. The US Angus shared similar family structure patterns with the AU Angus (i.e., animals on the right side vertical black line, Fig. 5) because similar genetic groups (represented by the colors) are observed in both countries.

Consistency of gametic phase measures the magnitude and the direction of the potential association between an SNP and a quantitative trait locus and is similar across populations. The consistency of gametic phase estimated between the US and AU populations was high (Fig. 4), indicating a potential benefit of performing joint genomic evaluations [48, 49].

### Genetic parameters

Heritability estimates for FA and CS across all models were similar and ranged from 0.21 to 0.27, regardless of the model and scenario used (Table 3 and Additional file 1: Tables S3–S6). In general, FA and CS for the AU population always presented slightly higher heritability estimates compared to the US population estimates. Furthermore, the majority of the scores from AU come from independent technicians (97% of records) rather than directly recorded by farmers as in the US, which as consequence, could have reduced the scoring bias and increased the variability of the scores. In spite of the small differences in dataset truncation and models, similar heritability estimates were observed for foot scores in our study and previously published studies in beef cattle (e.g., heritability estimates for FA range from 0.19 to 0.34, and for CS from 0.09 to 0.33 [2, 24–26]).

Wang et al. [25] used a subset (scores collected up to 2016) of the US dataset that was used in this study and they also truncated their data for single records collected on animals ranging in age from 320 to 460 days [25]. They reported heritability estimates of 0.37 for FA and 0.25 for CS in the US population [25]. Jeyaryban et al. [24] analyzed a subset (records up to 2008) of the AU dataset that was used in this study, but only included single records on animals younger than 750 days. The authors reported heritability estimates ranging from 0.29 to 0.32 for FA and from 0.29 to 0.33 for CS [24]. Jeyaryban et al. [24] considered the front and rear leg measurements of foot scores as different traits, and the genetic correlation between them was positive, favorable, and of moderate-to-high magnitude (0.87 for FA and 0.69 for CS in the AU population [18]). Considering that foot scores recorded on the front and rear legs are moderately to highly genetically correlated, both traits (front and rear foot scores) could be analyzed together, and one record (either front or rear leg) would be enough to capture the genetic variation and to select for improvements in foot score. Similar genetic correlations between front and rear leg foot scores were observed in North American Red Angus (0.88 for FA and 0.75 for CS; [2]). To optimize the data collection of foot scores, and as a consequence of the high genetic correlation between the front and rear leg foot scores, the AAA opted for using one record (front or rear leg) from each animal instead of two records [6, 7].

Foot scores (i.e., FA_US_ and CS_US_ or FA_AU_ and CS_AU_) are positive, favorable, and moderately-to-highly genetically correlated (from 0.46 to 0.50, Table 3). The genetic correlations between FA and CS reported in previous studies are of moderate-to-high magnitude and always favorable: ranging from 0.22 to 0.79 [2, 24–26]. As a consequence of the favorable genetic correlation within each country between FA and CS, a two-trait model could be beneficial for a possible increase in individual accuracy.

The fact that the genetic correlation for foot scores between US and AU differs from 1 could be due to potential GxE, differential data collection protocols, or to artifacts of the data. A possible justification for the artifacts of the data are the differences in scoring and magnitude of genetic linkage between the two countries based on pedigree. Moreover, there are historical differences in the generation of foot score data for genetic evaluation purposes in both populations. The AU Angus farmers started to select for foot score based on official genetic evaluations earlier than the US Angus farmers, which may have resulted in differences in genetic trends. However, the genetic trends for sires that are grouped based on birth year were similar for CS and FA between both countries (see Additional file 2: Figs. S5 and S6).

There are key differences in the scoring systems across countries, as indicated in “Methods” section. First, the AU system has two measurements of foot score, on the front and rear legs, while in the US foot score is assessed on the worst feet. However, in our analyses, an additional filtering was performed for the AU data, which provided information for the foot score on the worst feet, thus mimicking the US dataset. Second, the majority of the foot scores for the AU population are collected by trained and independent technicians, while in the US, farmers record foot score themselves. Only 3% of all AU records were collected by farmers (2047 records). High genetic correlations were observed between data collected by farmers and technicians for FA_AU_ (0.91) and CS_AU_ (0.85; see Additional file 1: Table S21). Van Vleck and Cundiff [50] who evaluated the genetic relationship between traits measured in males and females, concluded that a genetic correlation higher than 0.85 would be sufficient to assume a non-significant interaction between the two subsets of data. Furthermore, the trait distribution for the AU population is right-skewed compared to the US population. Thus, we performed an additional analysis of the genetic parameters by transforming the phenotypes to a Z-score within country. However, the genetic parameters obtained were the same as when using the data on the original scale.

The distribution of age differences of the animals scored (see Additional file 2: Fig. S1) could be another source of noise in the genetic correlation between the US and AU traits. Older animals are more likely to express extreme foot scores than younger animals. Therefore, a larger variation of the phenotypes was observed in the AU dataset. For instance, age does not have a significant impact on foot score for the AU population [best linear unbiased estimate (BLUE) for the covariable age equal to 0.000 for both FA and CS], while it has a considerable impact on that for the US population (coefficient equal to 0.001 and 0.002 for FA and CS, respectively). These findings highlight the importance of accounting for the distribution of age differences between the US and AU populations, e.g. including age nested within country when a single population is considered.

Genetic parameters were estimated using the pedigree-based relationship matrix. As aforementioned, 25 to 41% of the animals in the pedigree overlapped between the US and AU Angus populations. Based on pedigree, expected relationships can be calculated, which ignore Mendelian sampling—another source of genetic variation [51, 52]. In fact, full siblings have a theoretical relatedness equal to 0.50, while their genomic relationship can range from ~ 0.35 to ~ 0.65 for example [52]. To estimate the genetic parameters including genomic information, we randomly sampled 12,500 genotyped animals with phenotypic information from each country (similar to the ssGWAS). The genomic-relationship matrix from 25,000 genotyped animals was blended with the pedigree-based matrix to create the $\mathbf{H}$ matrix. The genetic parameters based on genomic information are presented in Additional file 1: Table S5, and were found to be within the same range as those based on the pedigree-based relationship matrix (see Additional file 1: Table S4).

Finally, deviations from 1 for the genetic correlation between two distinct environments can be explained by GxE. Some differences between the US and AU populations could be due to the magnitude of temperature, humidity, altitude, and management conditions (e.g., pastures versus feedlot). However, those differences could also be observed within each country. In a study conducted using the AU Angus dataset, depending on the trait being evaluated, reranking of sires was observed across states, such as, between Victoria and Queensland [13]. A genetic correlation equal to 0.65 was reported for intramuscular fat, a carcass ultrasound scan trait, between Angus bulls raised in Victoria and Queensland [13], which the authors explained as resulting from a possible age difference at measurement [13]. Similarly, GxE have been reported within the US for some beef cattle breeds. In US Angus cattle, a genetic correlation of 0.74 was observed when contrasting cattle raised in high and low altitudes [8]. Furthermore, other studies in Hereford [10] and Angus [12] cattle reported genetic correlations as low as 0.50 between environments within a country, although the authors concluded that in a traditional national evaluation, the rankings of the sires are, overall, likely acceptable given the challenges of providing multiple breeding values for multiple environments. Therefore, the magnitudes of the GxE across-country estimates found in this study are similar to those observed within country in other studies [10, 12].

### Genomic prediction of breeding values

The ultimate goal of this study was to identify the optimal statistical model to perform a multi-country genomic evaluation for foot score traits. A joint genomic evaluation across countries has many benefits, such as increasing the size of the genomic reference population, and making the published breeding values directly comparable across the countries involved, which facilitates the exchange of genetic material. Scenarios mimicking a within-country genomic evaluation were considered as a control and baseline comparison (i.e., WC-ST or WC-TT). Furthermore, two scenarios for a joint genomic prediction were tested including US and AU as two different populations (i.e., accounting for GxE; MC-ST or MC-TT) and US and AU as a single population (JC-ST or JC-TT). The last scenario could be more optimal because a single-scale breeding value would be available for both countries, more computationally feasible, and an additional encouragement to the standardization of the scoring protocols across countries. In national genomic evaluations for foot scores, FA and CS are currently fitted in two-trait models. If each country needs to be treated as a different population, a four-trait model (two countries and two traits considering their potential correlation—MC analyses) would be required.

In summary, FA and CS benefited from a joint genomic evaluation. Including information from another country improved the accuracy of genomic breeding values from 0.34 (WC-US evaluation) to 0.58 (MC or JC) and from 0.35 (WC-AU evaluation) to 0.56 (MC or JC) for FA and 0.44 (US) to 0.56 (MC) and from 0.48 (AU) to 0.58 (MC; Figs. 6 and 7) for CS. Furthermore, the joint genomic evaluation scenarios, multiple-country (MC) or joint-country (JC) analyses, provided breeding values with lower level and dispersion bias.

For FA, the joint genomic evaluation considering either US and AU as two distinct populations (MC) or as a single population (JC) provided lowly biased results, less over/underestimated GEBV, and more accurate estimates (Fig. 6) for both US and AU target animals compared to a WC evaluation. For CS, the JC analyses provided higher accuracies than the MC scenario. Considering CS in the AU target population, the MC model provided numerically better bias and dispersion outcomes (Fig. 7b and d). Factors influencing the predictivity ability of a genomic model include the heritability of the trait [47, 53], population parameters [47], the size of the reference population [47, 54, 55], and the link between reference and target population [56, 57].

As predicted by formulas, traits with a lower heritability require larger reference populations to estimate the accuracy as a function of the previously mentioned factors (e.g., formulae implemented in [58]). The accuracy relies heavily on the size of the reference population. Foot scores are in the low-to-moderate range of heritability estimates, therefore, the greater gain in accuracy in a joint genomic evaluation can be justified due to the two-fold increase in the reference population size. Furthermore, the impact of adding information from other countries depends on the magnitude of the correlation between countries: the higher is the correlation between countries, the greater would be the contribution in the genomic predictions [59]. US and AU are highly linked at the pedigree and genomic level (Figs. 3, 4 and 5), and the genetic correlations between the populations were moderate-to-high (0.61 for FA and 0.74 for CS).

Hayes et al. [14] reviewed many GxE studies in livestock and reported that there was a consensus that a genetic correlation lower than 0.80 between environments results in considerable re-ranking, and therefore, that modelling GxE was recommended [14]. However, the implementation of GxE models using a large national and international dataset can be time-consuming and computationally-intensive, and the availability of multiple environment-dependent (e.g., country-dependent) breeding values can create an additional challenge for breeders when selecting breeding bulls. With that said and considering the similar performance of both modelling or not GxE, a joint genomic evaluation for foot scores disregarding GxE could be a practical option (i.e., JC-ST or JC-TT analyses). Furthermore, having a single (G)EBV estimate per animal from both countries would be more practical and likely facilitates the exchange of genetic material across countries. Consequently, increased genetic gain within country and greater genetic diversity would be expected. As the size of the datasets for FA and CS in both countries increases, these analyses should be revisited. For instance, there might be greater benefits in fitting MC models.

In general, within-country genomic evaluation when analyzing both FA and CS together outperformed the predictability of a model fitting FA and CS, separately (Figs. 6 and 7), as expected due to the moderate-to-high genetic correlation between both traits. For FA, there was a gain in accuracy from 0.34 (single FA model) to 0.55 (two-trait model for FA and CS) for US and from 0.35 (single FA model) to 0.49 (two-trait model for FA and CS) for AU. However, when the US and AU populations were analyzed jointly (either MC or JC analyses), a small or no gain was observed compared to an independent analysis for foot score traits or when exploring the genetic correlation between FA and CS in a multiple-trait genomic evaluation. The latest can be explained by the sample size: the within-country dataset is limited for FA and CS, however, given the common structure of the US and AU populations, when the datasets are combined, the sample size adds up and increases the power of prediction. In other words, if the US and AU datasets are analyzed together, the two trait-model for FA and CS does not result in as much additional predictive power as when each dataset is analyzed individually. Similarly to a single-trait model for either FA or CS, JC (FA_US_ = 0.58, FA_AU_ = 0.55, CS_US_ = 0.59, and CS_AU_ = 0.61) resulted in similar results compared to the analyses taking GxE into account (MC; FA_US_ = 0.58, FA_AU_ = 0.57, CS_US_ = 0.62, CS_AU_ =0 0.61), thus holding the same conclusions as those presented for an independent trait analysis for foot score.

Finally, we also performed an across-country genomic evaluation (Figs. 6 and 7). As expected, due to the similar population structure and the shared history between US and AU, considerable predictability was observed when using data from one country to predict the breeding values of animals from another country, even given the GxE, and the differences in recording and production system. For instance, for FA, AU alone predicted US animals (across-country evaluation) with an accuracy of 0.44 compared to an accuracy of 0.55 obtained by the US predicting its own animals (within-country evaluation) while, US alone predicted AU animals with an accuracy of 0.46 compared to an accuracy of 0.49 obtained by AU predicting its own animals. In summary, the ability of using data from one country to predict relatively well the breeding values of animals from another country supports the moderate-to-high genetic correlations between US and AU and the common population structure. However, a combined analysis of both countries provides more accurate results. As previously indicated, these analyses should be revisited as the datasets from both countries become larger.

### Single-step genome-wide association analyses

Few SNPs were significantly associated with foot scores, and they overlapped between the US and AU populations. In general, more significant regions were identified for the AU population, which may be justified by a slightly larger reference population (see Additional file 1: Tables 17 and 19). However, all additional SNPs that were significantly associated with foot scores in the AU population but not significantly associated with foot scores in the US population had a relevant effect on the US foot score traits (Fig. 8). In other words, even though those SNPs did not achieve the “arbitrary boundary” of significance in the US population, the region had a significant impact on the trait because at least one marker within the region was significant and the remaining ones almost reached the significance threshold (Fig. 8).

Relevant candidate genes that could be involved in the biological mechanisms underlying FA and CS were found on BTA1 (Fig. 8). Four and six genes are located in the region of significant SNPs for FA and CS, respectively (Tables 4 and 5). Four common genes were identified for both FA and CS, including *FGF12* (*fibroblast growth factor 12*), *ATP13A5* (*ATPase 13A5*), *HRASLS* (*HRAS like suppressor*), and *ENSBTAG00000055130*. The fibroblast growth factor gene family is known to be linked to the biological regulation of, for example, articular cartilage [60, 61], and FGF-18 is an anabolic growth factor involved in articular cartilage repair [62].

In dairy cattle, BTA1 has been highlighted to harbor regions that are significantly associated with other hoof disorders [1, 63]. Suchocki et al. [1] analyzed three subjective pathological measurements of hoof disorders in two Austrian dairy cattle breeds and detected genomic regions on five chromosomes that have a role in hoof disorders, including BTA1 and BTA13 (e.g., *C13H20orf194* gene on BTA13 [1]). In our study, we also found BTA13 to be an important region for FA, in which a gene from a similar family was identified: *C13H20orf173* (Table 4).

On BTA13, the *GDF5* gene (*growth differentiation factor 5*) was identified as a candidate for both FA_TT.US_ and FA_TT.AU_. GDF5 is also known as cartilage-derived morphogenetic protein-1, and studies in mice and humans have highlighted its importance for skeletal development and repair [64–66]. Knockout experiments have shown that GDF5-deficient mice have a decreased bone mineral content and an abnormal bone structure (International Mouse Phenotyping Consortium, www.mousephenotype.org; accessed 18 August 2022). Therefore, the regions identified in our study are relevant candidates to better understand the mechanisms underlying foot scores in both the US and AU Angus populations.

## Conclusions

American (US) and Australian (AU) Angus cattle populations have similar genetic backgrounds and population structures. A joint genomic evaluation across both countries provided better predictivity ability compared to within-country evaluations. Although a genotype-by-environment interaction (GxE) was observed based on the genetic correlations between the US and AU foot scores, a single-trait model considering the US and AU populations as a single-population provided similar predictivity ability in comparison to modeling GxE. Considering these two populations as a single population for genomic evaluation provides advantages, such as easier implementation and a potential encouragement for the adoption of consistent measurement protocols across countries. Analyzing foot angle and claw set, separately or jointly (two-trait model), in a within-country evaluation had a significant impact on the performance of the genomic predictions. However, when both the US and AU datasets are analyzed together, no clear benefit was observed for the analysis of foot angle and claw set, separately or jointly. An across-country evaluation also indicated that the reference populations from one country can be used to predict breeding values in the other population but combining the data from both countries resulted in more accurate GEBV. Finally, foot scores have a polygenic genetic architecture and several important candidate genes were identified in this study. For both foot angle and claw set, BTA1 showed shared genomic regions, including a gene from the fibroblast growth factor gene family, which has been widely reported to affect cartilage repair. Another growth factor gene was identified on BTA13 (i.e., *GDF5*), which has been validated, in animal models, as having a role in skeletal development.

### Supplementary Information

**Additional file 1: Table S1.** Descriptive analyses of the raw dataset of foot scores for American (US) and Australian (AU) Angus populations. The dataset provided presents the number of records, frequency of foot angle and claw set scores, number of animals, number of herds, number of contemporary groups, and date of birth of the animals. **Table S2.** Variance explained by the first ten principal components using different datasets to create the genomic relationship matrix. Variance explained by the 10 principal components using the four data subsets. **Table S3.** Estimates of the heritability for foot angle (FA) and claw set (CS) using the American (AU) and Australian (AU) Angus datasets analyzed with a single-trait model within each country dataset. **Table S4.** Genetic parameters (heritability, repeatability, and genetic correlation) for foot angle (FA) and claw set (CS) based on multi-country two-trait models (MC-TT) between the American (AU) and Australian (AU) Angus populations. **Table S5.** Genetic parameters (heritability, repeatability, and genetic correlation) for foot angle (FA) or claw set (CS) using genomic information based on a multi-country single-trait model (MC-ST) between American (US) and Australian (AU) Angus populations. **Table S6.** Genetic parameters for foot angle (FA) and claw set (CS) based on joint-country two-trait model (JC-TT). **Table S7.** Accuracy, bias, and dispersion of the genomic prediction: within-country two-trait (WC) and across-country two-trait (AC) model using Australian Angus (AU) as the estimation set. Predictive ability from a forward validation using the linear regression method, including bias, dispersion, and accuracy for a forward validation in the AU animals (2019–2020) and across-country evaluation.**Table S8.** Accuracy, bias, and dispersion of the genomic prediction: within-country two-trait (JC) and across-country two-trait (AC) model using American Angus (US) as the estimation set. Predictive ability from a forward validation using the linear regression method, including bias, dispersion, and accuracy for a forward validation in the US animals (2020) and across-country evaluation. **Table S9.** Accuracy, bias, and dispersion of the genomic prediction based on multi-country two-trait models (MC-TT). Predictive ability from a forward validation using the linear regression method, including bias, dispersion, and accuracy for a forward validation. **Table S10.** Accuracy, bias, and dispersion of the genomic prediction based on joint-country two-trait models (JC-TT). Predictive ability from a forward validation using the linear regression method, including bias, dispersion, and accuracy for a forward validation. **Table S11.** Accuracy, bias, and dispersion of the single-trait (ST) genomic prediction scenarios within-country (WC-ST), multi-country (MC-ST), and joint countries (JC-ST). Predictive ability from a forward validation using the linear regression method, including bias, dispersion, and accuracy in scenarios using genomic (ssGBLUP) or not (BLUP). **Table S12.** Predictive ability for all genomic prediction scenarios for the Australian Angus target population (AU) presented by validation year: for single-trait within-country (WC-ST), multi-country (MC-ST), and joint-country (JC-ST) models. Predictive ability from a forward validation using the linear regression method, including bias, dispersion, and accuracy in scenarios using genomic (ssGBLUP). Results are presented for each birth year validation group in the AU population. **Table S13.** Average individual accuracy for the genomic prediction scenarios for foot angle based on single-trait models (-ST). **Table S14.** Average individual accuracy for the genomic prediction scenarios for claw set based on single-trait models (-ST). **Table S15.** Pearson and Spearman correlations of genomic estimated breeding values (GEBV) of proven sires (with more than 50 progeny with phenotypic records and raised in either one of the countries) across scenarios for foot angle based on single-trait within-country (WC-ST), multi-country (MC-ST), and joint countries (JC-ST) models. **Table S16.** Pearson and Spearman correlations of genomic estimated breeding values (GEBV) of proven sires (with more than 50 progeny with phenotypic records and raised in either one of the countries) across scenarios for claw set based on single-trait within-country (WC-ST), multi-country (MC-ST), and joint countries (JC-ST) models. **Table S17.** Significant SNPs associated with foot angle, and the genes located within a 100-kb genomic region down- and up-stream based on multi-country single-trait models (MC-ST). The list of genes name, gene Ensembl identification, gene position, the identification of the significant SNP, the population in which the SNPs were identified (US or AU), and the log (p-value). **Table S18.** Gene ontology terms and pathways in which the annotated genes for foot angle are involved based on multi-country single-trait models (MC-ST). **Table S19.** Significant SNPs associated with claw set, and the genes located within a 100-kb genomic region down- and up-stream based on multi-country single-trait models (MC-ST). The list of genes name, gene Ensembl identification, gene position, the identification of the significant SNP, the population in which the SNP were identified (US or AU), and the log (p-value). **Table S20.** Gene ontology terms and pathways in which the annotated genes for claw set are involved based on a multi-country single-trait model (MC-ST). **Table S21.** Genetic parameters (heritability, repeatability, and genetic correlations) between dataset collected by technicians and farmers in the Australian Angus dataset: two-recorder-trait considering the covariance between FA and CS equal to zero (-ST).

**Additional file 2: Figure S1.** Age distribution of the US (green) and AU (red) Angus populations. Each dot represents an animal. **Figure S2.** Cross-validation error for different K values in the admixture analyses. **Figure S3.** Admixture analysis for the US and AU populations (K = 2 and K = 6). **Figure S4.** QQ-plot of p-values for the genome-wide association for foot angle and claw set. Four sets of plots. A. Foot angle for American (US) Angus population; B. Foot angle for Australian (AU) Angus population; C. Claw set for American (US) Angus population; D. Claw set for Australian (AU) Angus population. Lambda is an inflation metric; lambda statistic should be close to 1 if the SNPs fall within the expected range of significance or greater than one if the observed p-values are more significant than expected. **Figure S5.** Genetic trends of foot angle and claw set for US and AU as an independent country. The results come from a single-trait single-country analysis. Furthermore, in this plot only sires were used (males with at least one progeny), and years with at least 100 individuals. **Figure S6.** Genetic trend of foot angle (FA) and claw set (CS) for the US and AU, separately. The results come from a single-trait single-country analysis, in which 51,146 genotyped animals were used for the US and 27,041 for the AU dataset (only genotyped animals with a direct impact on the solution of mixed model equations: phenotyped-genotyped animals and genotyped animals in the pedigree tracing back four generations). Furthermore, in this plot only sires were used (males with at least one progeny), and years with at least 100 individuals.

## Data Availability

The data supporting the results of this article are included within the article and in its Additional files. The raw data cannot be made available, as it is property of the American and Australian Angus cattle producers, and this information is commercially sensitive.
