# Supplementary material for: Across-country genetic and genomic analyses of foot score traits in American and Australian Angus cattle
Source: Genet Sel Evol. 2023 Nov 2;55:76. doi: 10.1186/s12711-023-00850-x (PMC10621155; doi:10.1186/s12711-023-00850-x)
Supplement: Supplementary file 1 — Additional file 1: Table S1. Descriptive analyses of the raw dataset of foot scores for American (US) and Australian (AU) Angus populations. The dataset provided presents the number of records, frequency of foot angle and claw set scores, number of animals, number of herds, number of contemporary groups, and date of birth of the animals. Table S2. Variance explained by the first ten principal components using different datasets to create the genomic relationship matrix. Variance explained by the 10 principal components using the four data subsets. Table S3. Estimates of the heritability for foot angle (FA) and claw set (CS) using the American (AU) and Australian (AU) Angus datasets analyzed with a single-trait model within each country dataset. Table S4. Genetic parameters (heritability, repeatability, and genetic correlation) for foot angle (FA) and claw set (CS) based on multi-country two-trait models (MC-TT) between the American (AU) and Australian (AU) Angus populations. Table S5. Genetic parameters (heritability, repeatability, and genetic correlation) for foot angle (FA) or claw set (CS) using genomic information based on a multi-country single-trait model (MC-ST) between American (US) and Australian (AU) Angus populations. Table S6. Genetic parameters for foot angle (FA) and claw set (CS) based on joint-country two-trait model (JC-TT). Table S7. Accuracy, bias, and dispersion of the genomic prediction: within-country two-trait (WC) and across-country two-trait (AC) model using Australian Angus (AU) as the estimation set. Predictive ability from a forward validation using the linear regression method, including bias, dispersion, and accuracy for a forward validation in the AU animals (2019–2020) and across-country evaluation.Table S8. Accuracy, bias, and dispersion of the genomic prediction: within-country two-trait (JC) and across-country two-trait (AC) model using American Angus (US) as the estimation set. Predictive ability from a forward validation using t [file 12711_2023_850_MOESM1_ESM.docx]

**ADDITIONAL FILE 1**

**Table S1** Descriptive analyses of the raw dataset of foot scores for American (US) and Australian (AU) Angus populations.

| **Trait** | **Foot Angle** | | **Claw Set** | |
| --- | --- | --- | --- | --- |
| **Country** | **US** | **AU** | **US** | **AU** |
| No. Records | 85,549 | 85,439 | 85,549 | 85,334 |
| Score distribution |  |  |  |  |
| 1 | 41 | 1 | 3 | 0 |
| 2 | 289 | 0 | 61 | 0 |
| 3 | 436 | 2 | 303 | 1 |
| 4 | 3,360 | 49 | 3,294 | 382 |
| 5 | 52,583 | 9,155 | 47,681 | 4,845 |
| 6 | 22,685 | 51,798 | 27,280 | 51,323 |
| 7 | 5,297 | 21,937 | 5,620 | 25,273 |
| 8 | 738 | 2,161 | 1,037 | 3,118 |
| 9 | 120 | 171 | 270 | 392 |
| No. Animals | 75,020 | 74,161 | 75,020 | 74,207 |
| No. Herds | 1161 | 195 | 1161 | 206 |
| No. CG | 6,074 | 11,970 | 6,074 | 12,124 |
| Min-Max Birth Date | 1997-2020 | 1990-2020 | 1997-2020 | 1990-2020 |

US: American Angus registered animals; AU: Australian Angus registered animals; CG: contemporary groups.

**Table S2** Variance explained by the first ten principal components using different datasets to create the genomic relationship matrix.

|  | **Cumulative variance explained by principal components (PC)** | | | |
| --- | --- | --- | --- | --- |
| **PC** | **All genotyped animals** | **Animals with genotype and phenotype** | **Randomly sampled 100K from each US and AU** | **Randomly sampled 10K from each US and AU** |
| 1 | 1.0 | 1.5 | 1.5 | 1.5 |
| 2 | 1.6 | 2.3 | 2.3 | 2.3 |
| 3 | 2.2 | 2.8 | 2.9 | 2.9 |
| 4 | 2.6 | 3.4 | 3.4 | 3.4 |
| 5 | 3.1 | 3.8 | 3.9 | 3.9 |
| 6 | 3.5 | 4.3 | 4.3 | 4.3 |
| 7 | 3.8 | 4.7 | 4.7 | 4.7 |
| 8 | 4.0 | 5.0 | 5.1 | 5.1 |
| 9 | 4.3 | 5.4 | 5.4 | 5.5 |
| 10 | 4.5 | 5.8 | 5.7 | 5.8 |

**Table S3** Heritability for foot angle (FA) and claw set (CS) analyzing American (AU) and Australian (AU) angus datasets analyzed with a single-trait model within each country dataset.

| **Components** | **US** | | **AU** | |
| --- | --- | --- | --- | --- |
|  | **Foot angle (FA_US_)** | **Claw set (CS_US_)** | **Foot angle (FA_AU_)** | **Claw set (CS_AU_)** |
| $h^{2}$ | 0.22 (0.01) | 0.22 (0.01) | 0.24 (0.01) | 0.26 (0.01) |

US: American Angus registered animals; AU: Australian Angus registered animals; ^1^Values between parentheses is the standard error, and $h^{2}$ is the heritability. The model used in this analysis was a single-trait model for either foot angle and claw set; these models were fitted separately to the US and AU datasets (FA_US_, CS_US_, FA_AU_ or CS_AU_).

**Table S4** Genetic parameters between American (US) and Australian (AU) Angus populations and foot angle (FA) and claw set (CS) based on multi-country two-trait models (MC-TT).

|  | FA _MC.US_ | FA _MC.AU_ | CS _MC.US_ | CS _MC.AU_ |
| --- | --- | --- | --- | --- |
| FA _MC.US_ | $h^{2}=$ 0.22 (0.01) |  |  |  |
| FA _MC.AU_ | $r_{g}=$0.61 (0.10) | $h^{2}=$ 0.24 (0.01) |  |  |
| CS _MC.US_ | $r_{g}=$0.50 (0.04) | $r_{g}=$0.29 (0.13) | $h^{2}=$0.22 (0.01) |  |
| CS _MC.AU_ | $r_{g}=$0.27 (0.13) | $r_{g}=$0.46 (0.03) | $r_{g}=$0.76 (0.07) | $h^{2}=$0.26 (0.01) |

US: American Angus registered animals; AU: Australian Angus registered animals; ^1^Values between parentheses are the standard error; $h^{2}$ is the heritability, $r_{g}$ is the genetic correlation. Model used is a multi-country two-trait model (MC referent in Table 2).

**Table S5** Genetic parameters between American (US) and Australian (AU) Angus populations of foot angle (FA) or claw set (CS) using genomic information based on a multi-country single-trait model (MC-ST).

|  | **FA** | | **Claw set** | |
| --- | --- | --- | --- | --- |
|  | **US** | **AU** | **US** | **AU** |
| **US** | $h^{2}=$0.18 |  | $h^{2}=$0.18 |  |
|  | $rep=$ 0.33 |  | $rep=$ 0.32 |  |
| **AU** | $r_{g}=$0.76 | $h^{2}=$0.24 | $r_{g}=$0.78 | $h^{2}=$0.25 |
|  |  | $rep=$ 0.31 |  | $rep=$ 0.35 |

US: American Angus animals; AU: Australian Angus animals; ^1^Values between parentheses are the standard error; $h^{2}$ is the heritability, $rep$ is the repeatability, and $r_{g}$ is the genetic correlation between US and AU. In total, 12,500 genotyped animals from each country that had phenotype were sampled to perform this analysis. In total, there were 25,000 genotyped animals.

**Table S6** Genetic parameters between foot angle (FA) and claw set (CS) based on joint-country two-trait model (JC-TT).

|  | FA | CS |
| --- | --- | --- |
| CS | $h^{2}=$ 0.24 (0.01) |  |
| FA | $r_{g}=$0.46 (0.02) | $h^{2}=$ 0.24 (0.01) |

US: American Angus registered animals; AU: Australian Angus registered animals; ^1^Values between parentheses are the standard error; $h^{2}$ is the heritability, and $r_{g}$ is the genetic correlation.

**Table S7** Accuracy, bias, and dispersion of the genomic prediction: within-country two-trait (WC) and across-country two-trait (AC) model using Australian Angus (AU) as the estimation set.

| **Model used** | **Validation set** | **Symbol** | **Birth-year** | **Bias** | **Dispersion** | **Accuracy** |
| --- | --- | --- | --- | --- | --- | --- |
| **WC** | **AU** | **FA** | **2019-2020** | 0.02 | 0.85 | 0.49 |
|  |  | **CS** | **2019-2020** | 0.13 | 0.80 | 0.44 |
| **AC** | **AU data to predict other countries** | | | | | |
|  | **US** | **FA** | **2020** | 0.01 | 0.85 | 0.44 |
|  | **CA** |  | **All (700)** | -0.01 | 0.77 | 0.41 |
|  | **US** | **CS** | **2020** | -0.01 | 0.85 | 0.50 |
|  | **CA** |  | **All (700)** | 0.02 | 0.76 | 0.42 |

**Table S8** Accuracy, bias, and dispersion of the genomic prediction: within-country two-trait (JC) and across-country two-trait (AC) model using American Angus (US) as the estimation set.

| **Model used** | **Validation set** | **Symbol** | **Birth-year** | **Bias** | **Dispersion** | **Accuracy** |
| --- | --- | --- | --- | --- | --- | --- |
| **WC** | **US** | **FA** | **2020** | -0.02 | 0.87 | 0.55 |
|  |  | **CS** | **2020** | -0.03 | 0.91 | 0.57 |
| **AC** | **US data to predict other countries** | | | | | |
|  | **AU** | **FA** | **2019-2020** | 0.01 | 0.76 | 0.46 |
|  | **CA** |  | **All (700)** | -0.02 | 0.80 | 0.43 |
|  | **AU** | **CS** | **2019-2020** | 0.08 | 0.79 | 0.49 |
|  | **CA** |  | **All (700)** | -0.01 | 0.85 | 0.47 |

**Table S9** Accuracy, bias, and dispersion of the genomic prediction based on multi-country two-trait models (MC-TT).

| **Validation set** | **Symbol** | **Birth-year** | **Bias** | **Dispersion** | **Accuracy** |
| --- | --- | --- | --- | --- | --- |
| **US** | **FA** | **2020** | 0.00 | 0.95 | 0.58 |
|  | **CS** | **2020** | 0.00 | 0.98 | 0.62 |
| **AU** | **FA** | **2019-2020** | 0.00 | 0.98 | 0.57 |
|  | **CS** | **2019-2020** | 0.00 | 0.95 | 0.61 |

**Table S10** Accuracy, bias, and dispersion of the genomic prediction based on joint-country two-trait models (JC-TT).

| **Validation set** | **Symbol** | **Birth-year** | **Bias** | **Dispersion** | **Accuracy** |
| --- | --- | --- | --- | --- | --- |
| **US** | **FA** | **2020** | 0.00 | 0.96 | 0.58 |
|  | **CS** | **2020** | 0.00 | 0.96 | 0.59 |
| **AU** | **FA** | **2019-2020** | 0.01 | 0.97 | 0.55 |
|  | **CS** | **2019-2020** | 0.03 | 0.95 | 0.61 |
| **AU and US data to predict other countries** | | | | | |
| **CA** | **FA** | **All (700)** | 0.00 | 0.90 | 0.48 |
| **CA** | **CS** | **All (700)** | 0.01 | 0.86 | 0.53 |

**Table S11** Accuracy, bias, and dispersion of the single-trait (ST) genomic prediction scenarios within-country (WC-ST), multi-country (MC-ST), and joint countries (JC-ST).

|  |  |  |  | **ssGBLUP** | | | **BLUP** | | |
| --- | --- | --- | --- | --- | --- | --- | --- | --- | --- |
| **Trait** | **Scenario** | **Estimation** | **Val** | **Bias** | **Dispersion** | **Accuracy** | **Bias** | **Dispersion** | **Accuracy** |
| **FA** | **WC-ST** | **US** | US | 0.007 | 1.342 | 0.336 | -0.041 | 0.581 | 0.336 |
|  | **WC-ST** | **AU** | AU | 0.000 | 1.087 | 0.346 | -0.042 | 0.873 | 0.299 |
|  | **MC-ST** | **US and AU** | US | 0.000 | 0.931 | 0.576 | 0.001 | 0.988 | 0.438 |
|  |  |  | AU | 0.007 | 0.966 | 0.559 | 0.011 | 0.975 | 0.328 |
|  | **JC-ST** | **US and AU** | US | 0.000 | 0.944 | 0.578 | 0.007 | 0.990 | 0.430 |
|  |  |  | AU | 0.003 | 0.977 | 0.555 | 0.008 | 0.971 | 0.329 |
| **CS** | **WC-ST** | **US** | US | 0.008 | 1.101 | 0.441 | -0.006 | 1.045 | 0.418 |
|  | **WC-ST** | **AU** | AU | -0.061 | 0.770 | 0.477 | -0.033 | 0.945 | 0.373 |
|  | **MC-ST** | **US and AU** | US | 0.008 | 1.051 | 0.559 | -0.011 | 1.120 | 0.476 |
|  |  |  | AU | 0.042 | 0.962 | 0.583 | -0.002 | 0.954 | 0.397 |
|  | **JC-ST** | **US and AU** | US | -0.008 | 0.988 | 0.586 | -0.034 | 1.113 | 0.471 |
|  |  |  | AU | 0.073 | 0.939 | 0.598 | -0.020 | 0.937 | 0.393 |

US: genomic prediction mimicking the within American Angus evaluation, in which just phenotype from American registered Angus were used; AU: genomic prediction mimicking the within Australian Angus evaluation, in which just phenotype from Australian registered Angus were used; USvsAU: joint-genomic evaluation between US and AU, but countries were considered as different traits (i.e., considering the genotype-by-environment interaction); USplusAU: joint-genomic prediction, considering US and AU as a single-population (i.e., single-trait). Val: subset the validation group by countries to compare them. ssGBLUP: single-step Genomic Best Linear Unbiased Prediction, genomic information is included. BLUP: pedigree-based relationship matrix, no genomic information was included.

**Table S12** Predictive ability for all genomic prediction scenarios for Australian Angus target population (AU) breakdown by validation year: for single-trait within-country (WC-ST), multi-country (MC-ST), and joint-country (JC-ST) models.

| **Trait** | **Scenario** | **Birth-year** | **Bias** | **Dispersion** | **Accuracy** |
| --- | --- | --- | --- | --- | --- |
| **FA** | **WC-ST** | 2020 | 0.002 | 1.227 | 0.324 |
|  |  | 2019 | 0.000 | 1.078 | 0.347 |
|  | **MC-ST** | 2020 | 0.011 | 0.991 | 0.579 |
|  |  | 2019 | 0.006 | 0.963 | 0.557 |
|  | **JC-ST** | 2020 | 0.005 | 0.994 | 0.592 |
|  |  | 2019 | 0.003 | 0.975 | 0.552 |
| **CS** | **WC-ST** | 2020 | -0.057 | 0.714 | 0.446 |
|  |  | 2019 | -0.062 | 0.774 | 0.480 |
|  | **MC-ST** | 2020 | 0.0432 | 0.945 | 0.543 |
|  |  | 2019 | 0.040 | 0.973 | 0.549 |
|  | **JC-ST** | 2020 | 0.070 | 0.947 | 0.576 |
|  |  | 2019 | 0.073 | 0.938 | 0.600 |

**Table S13** Average of individual (theoretical) accuracy for the genomic prediction scenarios for foot angle: single-trait models(-ST).

|  | **US** | | | | **AU** | | | |
| --- | --- | --- | --- | --- | --- | --- | --- | --- |
| **Scenarios** | **All ^2^** | **Pheno^3^** | **Sires^4^** | **Shared^5^** | **All** | **Pheno** | **Sires** | **Shared** |
| **Animals^1^** | 1,589,973 | 33,340 | 84,610 | 4,109 | 240,648 | 28,138 | 14,474 | 68 |
| **US WC-ST** | 0.12 (0.07) | 0.25 (0.04) | 0.10 (0.08) | 0.11 (0.14) | 0.06 (0.05) | 0.07 (0.04) | 0.05 (0.05) | 0.06 (0.05) |
| **AU WC-ST** | 0.08 (0.05) | 0.09 (0.04) | 0.07 (0.06) | 0.10 (0.13) | 0.14 (0.08) | 0.27 (0.07) | 0.15 (0.12) | 0.33 (0.24) |
| **US MC-ST** | 0.14 (0.07) | 0.26 (0.04) | 0.11 (0.09) | 0.12 (0.15) | 0.10 (0.05) | 0.14 (0.03) | 0.09 (0.06) | 0.14 (0.08) |
| **AU MC-ST** | 0.11 (0.06) | 0.15 (0.04) | 0.10 (0.07) | 0.11 (0.14) | 0.15 (0.09) | 0.28 (0.07) | 0.15 (0.13) | 0.33 (0.24) |
| **JC-ST** | 0.13 (0.07) | 0.26 (0.04) | 0.11 (0.09) | 0.14 (0.17) | 0.15 (0.08) | 0.28 (0.06) | 0.15 (0.12) | 0.33 (0.24) |

^1^Animals: Number of animals. All the scenarios would share animals due to the common genotyped information. However, the number of phenotype information changed accordingly its scenario, as shown in Table 2 (main manuscript). ^2^All: All animals in which genomic breeding values are being calculated, which includes animals in the pedigree, phenotype, and genotyped files. ^3^Pheno: Animals registered in that specific country which originally have phenotype for the trait; however, the value is lower than presented in Table 2 (main manuscript), because animals with phenotype, but for which the breeding values were estimated in other scenarios were kept for comparisons. ^4^Sires: registered sires (i.e., at least one progeny) for each country. ^5^Shared: animals in the pedigree overlapping between US and AU, animals that would have progenies in both countries. Average of individual accuracy was calculated as Beef Improvement Federation’s recommendation.

**Table S14** Average of individual (theoretical) accuracy for the genomic evaluation scenarios considering claw set: single-trait models (-ST).

|  | **US** | | | | **AU** | | | |
| --- | --- | --- | --- | --- | --- | --- | --- | --- |
| **Scenarios** | **All ^2^** | **Pheno^3^** | **Sires^4^** | **Shared^5^** | **All** | **Pheno** | **Sires** | **Shared** |
| **Animals^1^** | 1,589,973 | 35,481 | 84,610 | 4,109 | 240,648 | 28,311 | 14,474 | 68 |
| **US WC-ST** | 0.12 (0.07) | 0.25 (0.04) | 0.10 (0.08) | 0.11 (0.14) | 0.06 (0.05) | 0.06 (0.04) | 0.05 (0.05) | 0.05 (0.05) |
| **AU WC-ST** | 0.09 (0.05) | 0.10 (0.04) | 0.08 (0.06) | 0.10 (0.13) | 0.14 (0.09) | 0.29 (0.06) | 0.15 (0.13) | 0.33 (0.24) |
| **US MC-ST** | 0.14 (0.07) | 0.27 (0.04) | 0.12 (0.09) | 0.13 (0.15) | 0.12 (0.06) | 0.19 (0.03) | 0.11 (0.08) | 0.19 (0.11) |
| **AU MC-ST** | 0.13 (0.07) | 0.20 (0.04) | 0.11 (0.08) | 0.13 (0.15) | 0.16 (0.09) | 0.30 (0.06) | 0.17 (0.13) | 0.34 (0.24) |
| **JC-ST** | 0.14 (0.07) | 0.27 (0.04) | 0.12 (0.09) | 0.14 (0.17) | 0.15 (0.09) | 0.29 (0.06) | 0.15 (0.13) | 0.33 (0.24) |

Descriptions as presented in Table S10.

**Table S15** Pearson and Spearman correlations of genomic estimated breeding values (GEBV) of proved sires (with more than 50 progeny with phenotypic records and raised in either one of the countries) across scenarios for foot angle based on single-trait within-country (WC-ST), multi-country (MC-ST), and joint countries (JC-ST) models.

| Sires’ origin | Models | N# Sires | Pearson | Spearman |
| --- | --- | --- | --- | --- |
| US | WC-ST vs MC-ST | 3,073 | 0.84 | 0.83 |
|  | WC-STvs JC-ST |  | 0.75 | 0.73 |
|  | MC-ST vs JC-ST |  | 0.95 | 0.94 |
| AU | WC-ST vs MC-ST | 604 | 0.92 | 0.91 |
|  | WC-ST vs JC-ST |  | 0.91 | 0.89 |
|  | MC-ST vs JC-ST |  | 0.98 | 0.98 |

**Table S16** Pearson and Spearman correlations of genomic estimated breeding values (GEBV) of proved sires (with more than 50 progeny with phenotypic records and raised in either one of the countries) across scenarios for claw set based on single-trait within-country (WC-ST), multi-country (MC-ST), and joint countries (JC-ST) models.

| Sires’ origin | Models | N# Sires | Pearson | Spearman |
| --- | --- | --- | --- | --- |
| US | WC-ST vs MC-ST | 3,073 | 0.87 | 0.86 |
|  | WC-ST vs JC-ST |  | 0.78 | 0.77 |
|  | MC-ST vs JC-ST |  | 0.96 | 0.96 |
| AU | WC-ST vs MC-ST | 602 | 0.92 | 0.92 |
|  | WC-ST vs JC-ST |  | 0.90 | 0.89 |
|  | MC-ST vs JC-ST |  | 0.99 | 0.99 |

**Table S17** Significant SNPs associated with foot angle, and the genes located within a genomic region of 100 kilobases down and upstream based on multi-country single-trait models (MC-ST).

| Chr | Gene name | Gene Ensembl | Gene position | SNP rs ID | SNP position | Population | -log10(P-value) |
| --- | --- | --- | --- | --- | --- | --- | --- |
| 1 | *FGF12* | ENSBTAG00000012413 | 74876548:75283017 | rs109401728 | 74893925 | US and AU | US:4.81 and AU:5.62 |
| 1 | *ATP13A5* | ENSBTAG00000018657 | 74043235:74159562 | rs41630299 | 74141004 | AU | 4.59 |
| 1 | *HRASLS* | ENSBTAG00000035844 | 74163666:74183456 | rs41630299 | 74141004 | AU | 4.59 |
| 1 | *bta-mir-10182* | ENSBTAG00000055130 | 74829775:74829854 | rs109401728 | 74893925 | US and AU | US:4.81 and AU:5.62 |
| 5 |  |  |  | rs109643119 | 18519042 | US and AU | US:4.79 and AU:4.06 |
| 13 | *TRPC4AP* | ENSBTAG00000002236 | 64304949:64374165 | rs43716526 | 64312628 | AU | 4.09 |
| 13 | *TRPC4AP* | ENSBTAG00000002236 | 64304949:64374165 | rs43717453 | 64232510 | AU | 4.63 |
| 13 | *GSS* | ENSBTAG00000003504 | 64234188:64259465 | rs43716526 | 64312628 | AU | 4.09 |
| 13 | *GSS* | ENSBTAG00000003504 | 64234188:64259465 | rs43717453 | 64232510 | AU | 4.63 |
| 13 | *GSS* | ENSBTAG00000003504 | 64234188:64259465 | rs43717461 | 64196996 | AU | 4.63 |
| 13 | *MYH7B* | ENSBTAG00000003512 | 64280737:64309631 | rs43716526 | 64312628 | AU | 4.09 |
| 13 | *MYH7B* | ENSBTAG00000003512 | 64280737:64309631 | rs43717453 | 64232510 | AU | 4.63 |
| 13 | *MYH7B* | ENSBTAG00000003512 | 64280737:64309631 | rs43717461 | 64196996 | AU | 4.63 |
| 13 | *EDEM2* | ENSBTAG00000003815 | 64392791:64420836 | rs110256558 | 64505651 | AU | 4.19 |
| 13 | *EDEM2* | ENSBTAG00000003815 | 64392791:64420836 | rs43716526 | 64312628 | AU | 4.09 |
| 13 | *RBM39* | ENSBTAG00000003949 | 64908243:64934850 | rs43711225 | 64812028 | AU | 4.49 |
| 13 | *GDF5* | ENSBTAG00000004429 | 64681475:64685560 | rs43711168 | 64765574 | US and AU | US:4.23 and AU:5.14 |
| 13 | *CEP250* | ENSBTAG00000006021 | 64699831:64746870 | rs110042294 | 64787127 | US and AU | AU:4.46 and AU:4.99 |
| 13 | *CEP250* | ENSBTAG00000006021 | 64699831:64746870 | rs43711168 | 64765574 | US and AU | US:4.23 and AU:5.14 |
| 13 | *CEP250* | ENSBTAG00000006021 | 64699831:64746870 | rs43711225 | 64812028 | AU | 4.49 |
| 13 | *FAM83C* | ENSBTAG00000006278 | 64554623:64561144 | rs110254892 | 64541923 | AU | 4.63 |
| 13 | *FAM83C* | ENSBTAG00000006278 | 64554623:64561144 | rs110256558 | 64505651 | AU | 4.19 |
| 13 | *ERGIC3* | ENSBTAG00000006670 | 64776869:64791647 | rs110042294 | 64787127 | US and AU | AU:4.46 and AU:4.99 |
| 13 | *ERGIC3* | ENSBTAG00000006670 | 64776869:64791647 | rs43711168 | 64765574 | US and AU | US:4.23 and AU:5.14 |
| 13 | *ERGIC3* | ENSBTAG00000006670 | 64776869:64791647 | rs43711225 | 64812028 | AU | 4.49 |
| 13 | *SPAG4* | ENSBTAG00000006949 | 64841049:64845754 | rs110042294 | 64787127 | US and AU | AU:4.46 and AU:4.99 |
| 13 | *SPAG4* | ENSBTAG00000006949 | 64841049:64845754 | rs43711168 | 64765574 | US and AU | US:4.23 and AU:5.14 |
| 13 | *SPAG4* | ENSBTAG00000006949 | 64841049:64845754 | rs43711225 | 64812028 | AU | 4.49 |
| 13 | *CPNE1* | ENSBTAG00000006955 | 64848971:64884882 | rs110042294 | 64787127 | US and AU | AU:4.46 and AU:4.99 |
| 13 | *CPNE1* | ENSBTAG00000006955 | 64848971:64884882 | rs43711168 | 64765574 | US and AU | US:4.23 and AU:5.14 |
| 13 | *CPNE1* | ENSBTAG00000006955 | 64848971:64884882 | rs43711225 | 64812028 | AU | 4.49 |
| 13 | *NFS1* | ENSBTAG00000006962 | 64887674:64904921 | rs43711225 | 64812028 | AU | 4.49 |
| 13 | *NCOA6* | ENSBTAG00000007930 | 64051984:64148363 | rs43717453 | 64232510 | AU | 4.63 |
| 13 | *NCOA6* | ENSBTAG00000007930 | 64051984:64148363 | rs43717461 | 64196996 | AU | 4.63 |
| 13 | *PROCR* | ENSBTAG00000008291 | 64444427:64449124 | rs110254892 | 64541923 | AU | 4.63 |
| 13 | *PROCR* | ENSBTAG00000008291 | 64444427:64449124 | rs110256558 | 64505651 | AU | 4.19 |
| 13 | *MMP24* | ENSBTAG00000010024 | 64512071:64543097 | rs110254892 | 64541923 | AU | 4.63 |
| 13 | *MMP24* | ENSBTAG00000010024 | 64512071:64543097 | rs110256558 | 64505651 | AU | 4.19 |
| 13 | *EIF6* | ENSBTAG00000011263 | 64544773:64553147 | rs110254892 | 64541923 | AU | 4.63 |
| 13 | *EIF6* | ENSBTAG00000011263 | 64544773:64553147 | rs110256558 | 64505651 | AU | 4.19 |
| 13 | *GGT7* | ENSBTAG00000013301 | 64158906:64183221 | rs43717453 | 64232510 | AU | 4.63 |
| 13 | *GGT7* | ENSBTAG00000013301 | 64158906:64183221 | rs43717461 | 64196996 | AU | 4.63 |
| 13 | *ACSS2* | ENSBTAG00000013303 | 64186743:64233568 | rs43716526 | 64312628 | AU | 4.09 |
| 13 | *ACSS2* | ENSBTAG00000013303 | 64186743:64233568 | rs43717453 | 64232510 | AU | 4.63 |
| 13 | *ACSS2* | ENSBTAG00000013303 | 64186743:64233568 | rs43717461 | 64196996 | AU | 4.63 |
| 13 | *ROMO1* | ENSBTAG00000027361 | 64904969:64906500 | rs43711225 | 64812028 | AU | 4.49 |
| 13 | *bta-mir-499* | ENSBTAG00000029949 | 64292583:64292680 | rs43716526 | 64312628 | AU | 4.09 |
| 13 | *bta-mir-499* | ENSBTAG00000029949 | 64292583:64292680 | rs43717453 | 64232510 | AU | 4.63 |
| 13 | *bta-mir-499* | ENSBTAG00000029949 | 64292583:64292680 | rs43717461 | 64196996 | AU | 4.63 |
| 13 | *C13H20orf173* | ENSBTAG00000030976 | 64765618:64768195 | rs110042294 | 64787127 | US and AU | AU:4.46 and AU:4.99 |
| 13 | *C13H20orf173* | ENSBTAG00000030976 | 64765618:64768195 | rs43711168 | 64765574 | US and AU | US:4.23 and AU:5.14 |
| 13 | *C13H20orf173* | ENSBTAG00000030976 | 64765618:64768195 | rs43711225 | 64812028 | AU | 4.49 |
| 13 | *UQCC1* | ENSBTAG00000030990 | 64573005:64669659 | rs110254892 | 64541923 | AU | 4.63 |
| 13 | *UQCC1* | ENSBTAG00000030990 | 64573005:64669659 | rs110256558 | 64505651 | AU | 4.19 |
| 13 | *UQCC1* | ENSBTAG00000030990 | 64573005:64669659 | rs43711168 | 64765574 | US and AU | US:4.23 and AU:5.14 |
| 13 | *RBM12* | ENSBTAG00000044015 | 64873751:64884908 | rs110042294 | 64787127 | US and AU | AU:4.46 and AU:4.99 |
| 13 | *RBM12* | ENSBTAG00000044015 | 64873751:64884908 | rs43711225 | 64812028 | AU | 4.49 |
| 13 |  | ENSBTAG00000052250 | 64681635:64682588 | rs43711168 | 64765574 | US and AU | US:4.23 and AU:5.14 |
| 13 |  | ENSBTAG00000053187 | 64792559:64837197 | rs110042294 | 64787127 | US and AU | AU:4.46 and AU:4.99 |
| 13 |  | ENSBTAG00000053187 | 64792559:64837197 | rs43711168 | 64765574 | US and AU | US:4.23 and AU:5.14 |
| 13 |  | ENSBTAG00000053187 | 64792559:64837197 | rs43711225 | 64812028 | AU | 4.49 |
| 13 |  | ENSBTAG00000053266 | 64573385:64573934 | rs110254892 | 64541923 | AU | 4.63 |
| 13 |  | ENSBTAG00000053266 | 64573385:64573934 | rs110256558 | 64505651 | AU | 4.19 |
| 13 |  | ENSBTAG00000053775 | 64908286:64908468 | rs43711225 | 64812028 | AU | 4.49 |
| 20 | *CREBRF* | ENSBTAG00000002020 | 4776513:4831767 | rs43350564 | 4709731 | US and AU | US:4.07 and AU:4.54 |
| 20 | *RPL26L1* | ENSBTAG00000015099 | 4716985:4723653 | rs43350564 | 4709731 | US and AU | US:4.07 and AU:4.54 |
| 20 | *ATP6V0E1* | ENSBTAG00000015100 | 4732524:4762537 | rs43350564 | 4709731 | US and AU | US:4.07 and AU:4.54 |
| 20 | *ERGIC1* | ENSBTAG00000015955 | 4596395:4709455 | rs43350564 | 4709731 | US and AU | US:4.07 and AU:4.54 |

**Table S18** Gene ontology terms and pathways in which the annotated genes for foot angle are involved based on multi-country single-trait models (MC-ST).

| Category | Term | Genes | P-value | Benjamini |
| --- | --- | --- | --- | --- |
| GO BP | Glutathione biosynthetic process (GO:0006750) | ENSBTAG00000013301, ENSBTAG00000003504 | 0.013 | 1 |
| GO BP | Response to unfolded protein (GO:0006986) | ENSBTAG00000003815, ENSBTAG00000002020 | 0.04 | 1 |
| GO BP | Ribosomal large subunit biogenesis (GO:0042273) | ENSBTAG00000015099, ENSBTAG00000011263 | 0.05 | 1 |
| GO BP | Retrograde vesicle-mediated transport, Golgi to ER (GO:0006890) | ENSBTAG00000015955, ENSBTAG00000006670 | 0.07 | 1 |
| KEGG | Glutathione metabolism (bta00480) | ENSBTAG00000013301, ENSBTAG00000003504 | 0.07 | 1 |
| KEGG | Metabolic pathways (bta01100) | ENSBTAG00000013301, ENSBTAG00000013303, ENSBTAG00000003504, ENSBTAG00000006962, ENSBTAG00000015100 | 0.09 | 1 |

Benjamin is a multiple comparison correction of the p-values, in which the p-values are adjusted based on the linear step-up method of Benjamin and Hochberg (1995).

**Table S19** Significant SNPs associated with claw set, and the genes located within a genomic region of 100 kilobases down and upstream based on multi-country single-trait models (MC-ST).

| Chr | Gene name | Gene Ensembl | Gene position | SNP rs ID | SNP position | Population | -log10(P-value) |
| --- | --- | --- | --- | --- | --- | --- | --- |
| 1 | *MB21D2* | ENSBTAG00000005430 | 74489024:74609955 | rs109316834 | 74688985 | US and AU | US:6.28 and AU:7.31 |
| 1 | *FGF12* | ENSBTAG00000012413 | 74876548:75283017 | rs109401728 | 74893925 | US and AU | US:4.85 and AU:6.37 |
| 1 | *ATP13A5* | ENSBTAG00000018657 | 74043235:74159562 | rs41630299 | 74141004 | US and AU | US:5.74 and AU:7.03 |
| 1 | *PLAAT1* | ENSBTAG00000035844 | 74163666:74183456 | rs41630299 | 74141004 | US and AU | US:5.74 and AU:7.03 |
| 1 |  | ENSBTAG00000053503 | 74675757:74698158 | rs109316834 | 74688985 | US and AU | US:6.28 and AU:7.31 |
| 1 | *bta-mir-10182* | ENSBTAG00000055130 | 74829775:74829854 | rs109401728 | 74893925 | US and AU | US:4.85 and AU:6.37 |
| 5 |  |  |  | rs109643119 | 18519042 | US and AU | US:5.41 and AU:5.15 |
| 5 |  |  |  | rs29012239 | 18679362 | AU | 4.26 |
| 5 | *KITLG* | ENSBTAG00000017549 | 18250809:18353485 | rs41654028 | 18388982 | AU | 4.92 |
| 11 | *FUBP3* | ENSBTAG00000013072 | 100882266:100931850 | rs109998709 | 100903015 | AU | 4.39 |
| 11 | *ABL1* | ENSBTAG00000017976 | 100988747:101131037 | rs109998709 | 100903015 | AU | 4.39 |
| 11 | *ASS1* | ENSBTAG00000020747 | 100770166:100822252 | rs109998709 | 100903015 | AU | 4.39 |
| 11 | *PRDM12* | ENSBTAG00000023797 | 100951292:100966190 | rs109998709 | 100903015 | AU | 4.39 |
| 11 | *U6* | ENSBTAG00000043722 | 100937424:100937524 | rs109998709 | 100903015 | AU | 4.39 |
| 11 | *EXOSC2* | ENSBTAG00000048071 | 100975458:100985125 | rs109998709 | 100903015 | AU | 4.39 |
| 19 |  |  |  | rs41640388 | 59702745 | US and AU | US:4.41 and AU:4.84 |
| 23 | *SUPT3H* | ENSBTAG00000032887 | 18239634:18641106 | rs41618604 | 18732494 | US and AU | US:5.25 and AU:5.00 |

**Table S20** Gene ontology terms and pathways in which the annotated genes for claw set are involved based on a multi-country single-trait model (MC-ST).

| Category | Term | Genes | P-value | Benjamini |
| --- | --- | --- | --- | --- |
| GO BP | Positive regulation of peptidyl-tyrosine phosphorylation (GO:0050731) | ENSBTAG00000017549, ENSBTAG00000017976 | 0.03 | 1 |
| GO BP | Regulation of gene expression (GO:0010468) | ENSBTAG00000013072, ENSBTAG00000023797 | 0.09 | 1 |
| GO MF | GO:0008083~growth factor activity | ENSBTAG00000017549, ENSBTAG00000012413 | 0.07 | 1 |

Benjamini is a multiple comparison correction of the p-values, in which the p-values are adjusted based on the linear step-up method of Benjamini and Hochberg (1995).

**Table S21** Genetic parameters for foot angle and claw set based on datasets collected by technicians and farmers in the Australian Angus dataset: two-recorder-trait considering the covariance between FA and CS equal to zero (-ST).

|  | **Foot angle** | | **Claw set** | |
| --- | --- | --- | --- | --- |
|  | **Technician** | **Farmer** | **Technician** | **Farmer** |
|  | 68,532 | 1,932 | 68,894 | 2,015 |
| Technician | $h^{2}=$0.24 (0.01) |  | $h^{2}=$0.26 (0.01) |  |
|  | $rep=$ 0.32 (0.01) |  | $rep=$ 0.35 (0.01) |  |
| Farmer | $r_{g}=$0.91 (0.07) | $h^{2}=$0.32 (0.05) | $r_{g}=$0.85 (0.08) | $h^{2}=$0.32 (0.05) |

^1^Values between parentheses is the standard error; $h^{2}$ is the heritability, $rep$ is the repeatability, and $r_{g}$ is the genetic correlation between foot score measured by a technician and farmer.
