# Supplementary material for: Across-country genetic and genomic analyses of foot score traits in American and Australian Angus cattle
Source: Genet Sel Evol. 2023 Nov 2;55:76. doi: 10.1186/s12711-023-00850-x (PMC10621155; doi:10.1186/s12711-023-00850-x)
Supplement: Supplementary file 2 — Additional file 2: Figure S1. Age distribution of the US (green) and AU (red) Angus populations. Each dot represents an animal. Figure S2. Cross-validation error for different K values in the admixture analyses. Figure S3. Admixture analysis for the US and AU populations (K = 2 and K = 6). Figure S4. QQ-plot of p-values for the genome-wide association for foot angle and claw set. Four sets of plots. A. Foot angle for American (US) Angus population; B. Foot angle for Australian (AU) Angus population; C. Claw set for American (US) Angus population; D. Claw set for Australian (AU) Angus population. Lambda is an inflation metric; lambda statistic should be close to 1 if the SNPs fall within the expected range of significance or greater than one if the observed p-values are more significant than expected. Figure S5. Genetic trends of foot angle and claw set for US and AU as an independent country. The results come from a single-trait single-country analysis. Furthermore, in this plot only sires were used (males with at least one progeny), and years with at least 100 individuals. Figure S6. Genetic trend of foot angle (FA) and claw set (CS) for the US and AU, separately. The results come from a single-trait single-country analysis, in which 51,146 genotyped animals were used for the US and 27,041 for the AU dataset (only genotyped animals with a direct impact on the solution of mixed model equations: phenotyped-genotyped animals and genotyped animals in the pedigree tracing back four generations). Furthermore, in this plot only sires were used (males with at least one progeny), and years with at least 100 individuals. [file 12711_2023_850_MOESM2_ESM.docx]

**ADDITIONAL FILE 2**


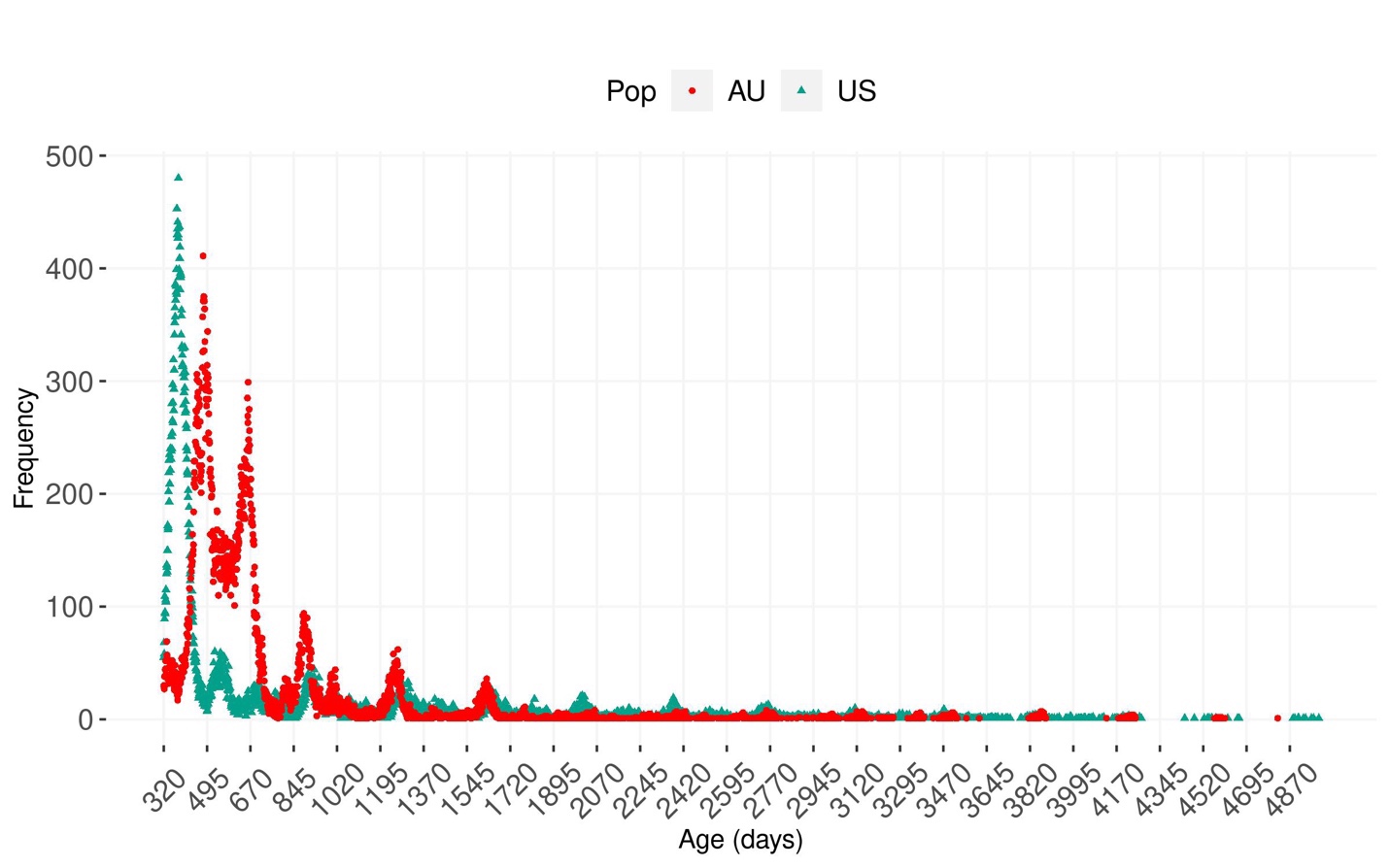


**Figure S1** Age distribution of US (green) and AU (red) Angus populations.

**
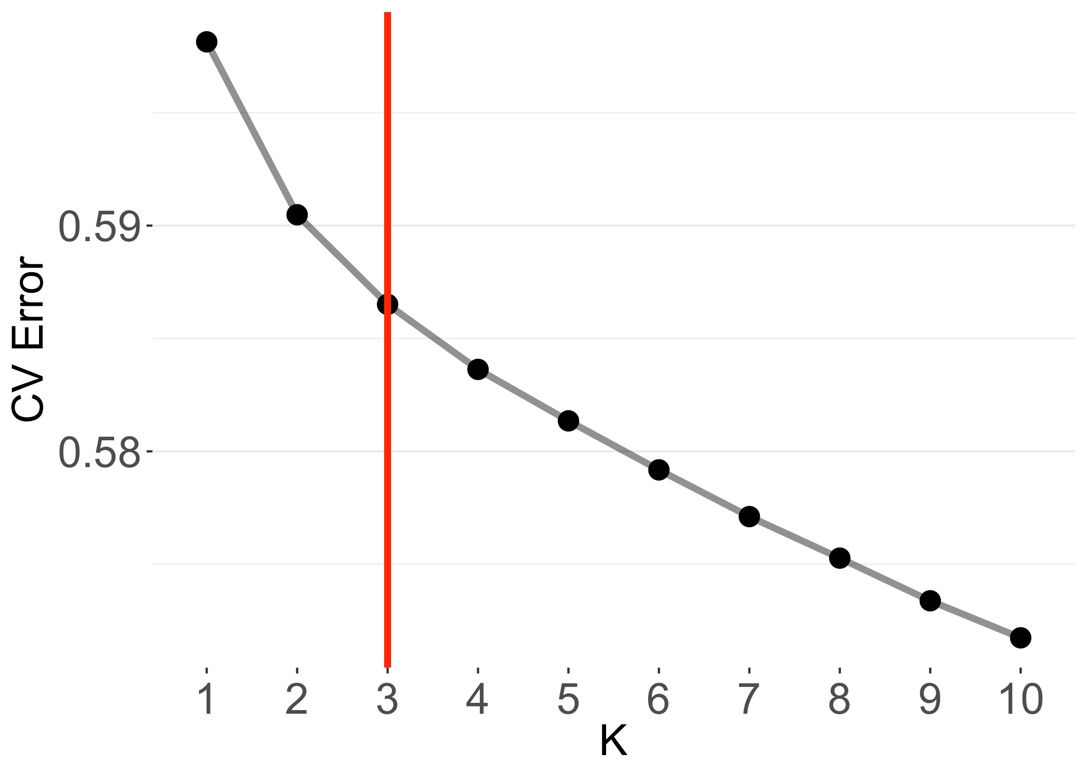
**

**Figure S2** Cross-validation error for different K values in the ADMIXTURE analyses.


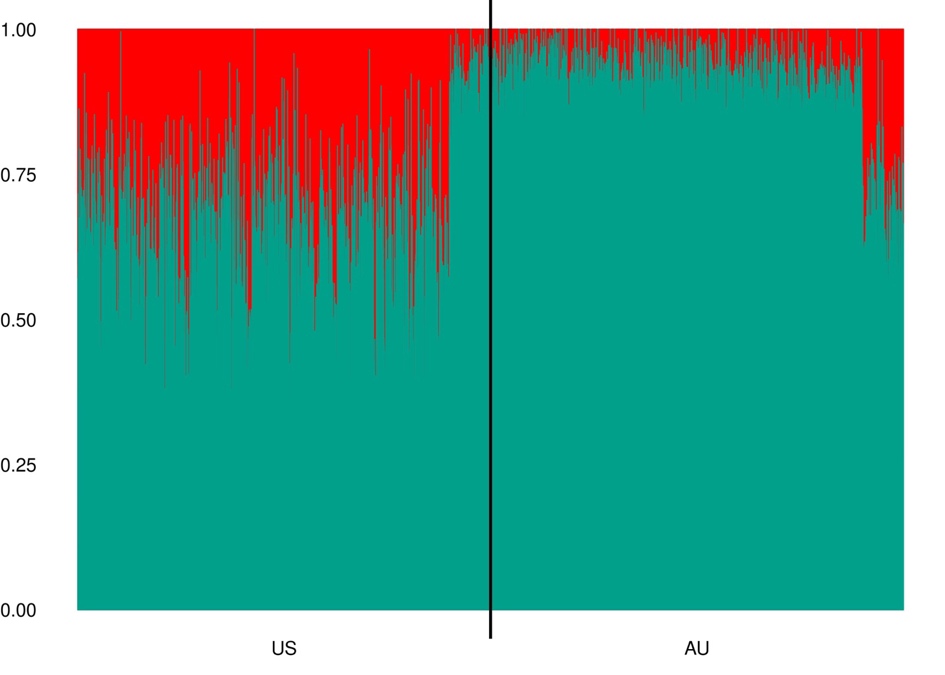

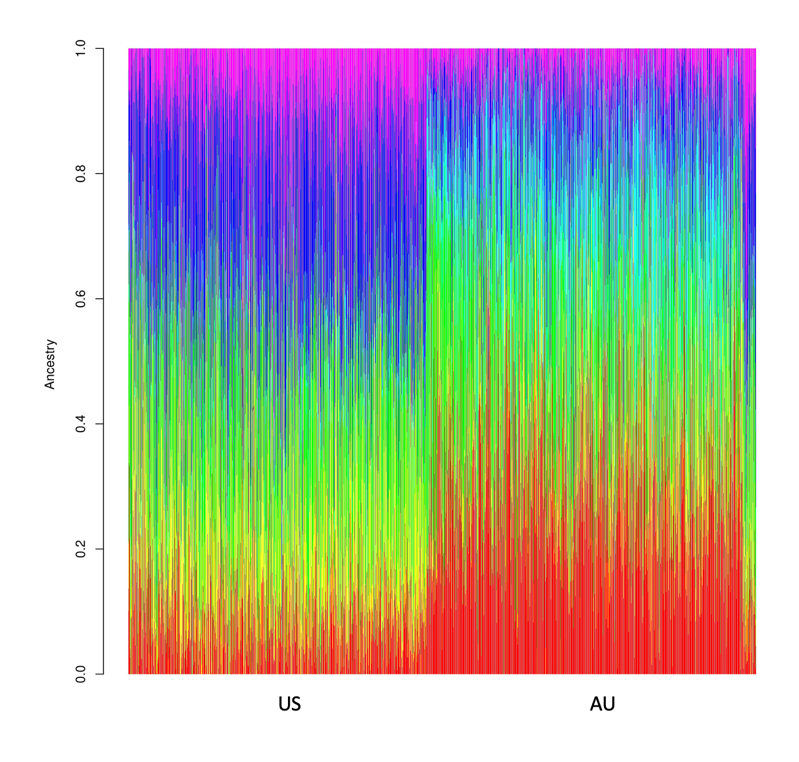


**Figure S3** Admixture analysis for US and AU populations (top K = 2, bottom K = 6).


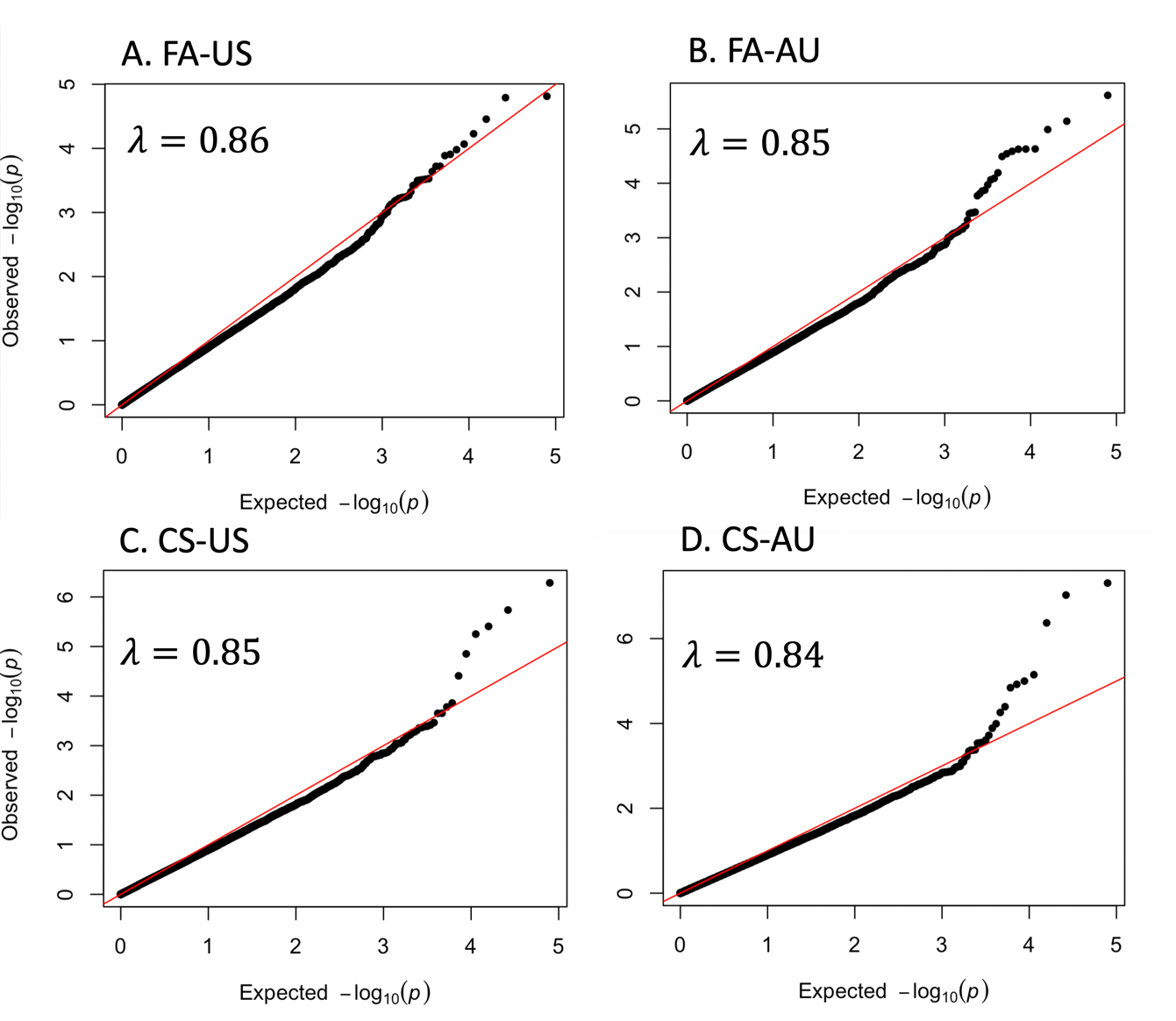


**Figure S4** QQ-plot of p-values for the genome-wide association for foot angle and claw set.

Lambda is an inflation metric; lambda statistic should be close to 1 if the SNPs fall within the expected range of significancy or greater than one if the observed p-values are more significant than expected.


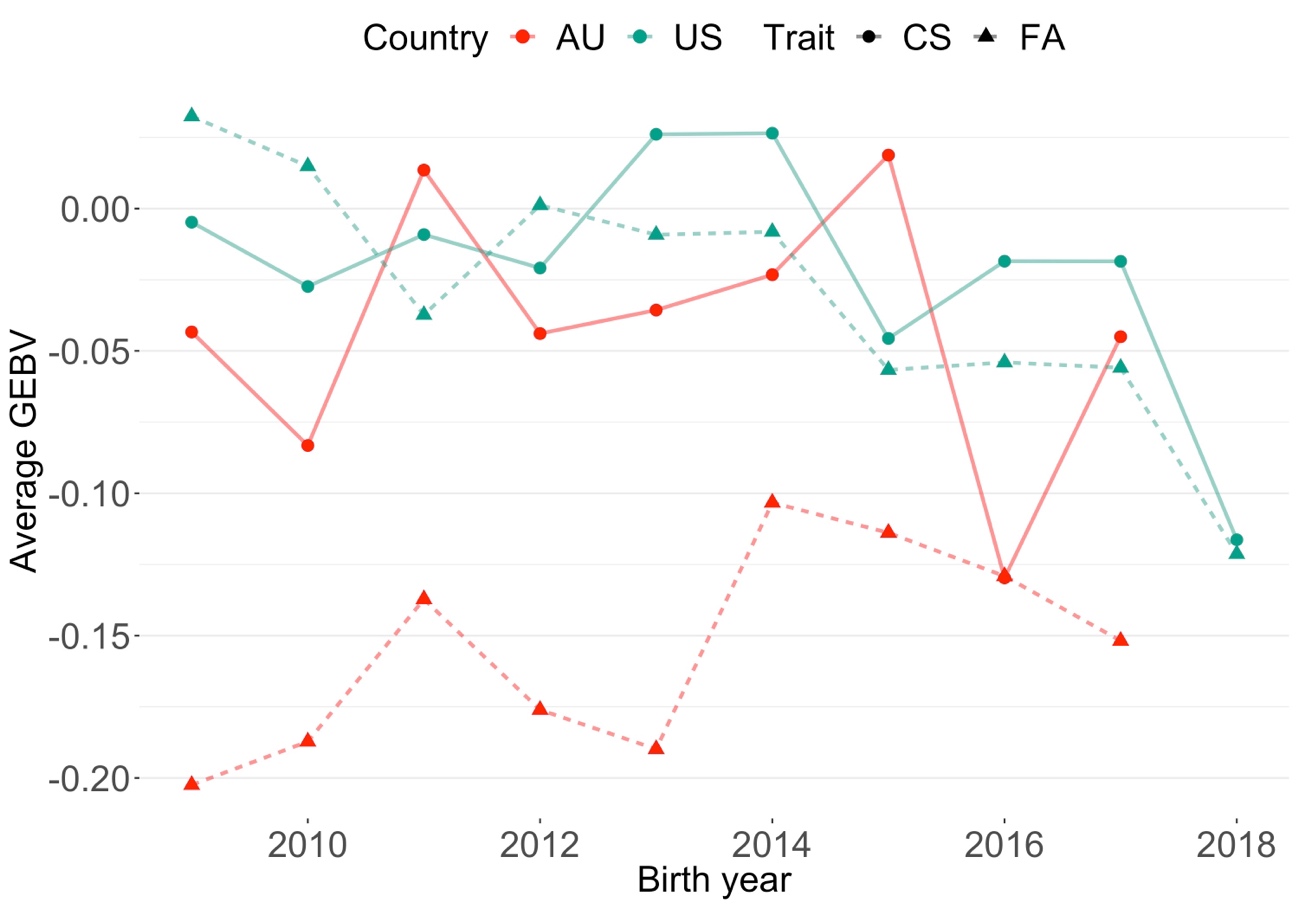


**Figure S5** Genetic trends of foot angle and claw set for US and AU as an independent country. The results come from a single-trait single-country analysis. Furthermore, in this plot only sires were used (males with at least one progeny), and years with at least 100 individuals.


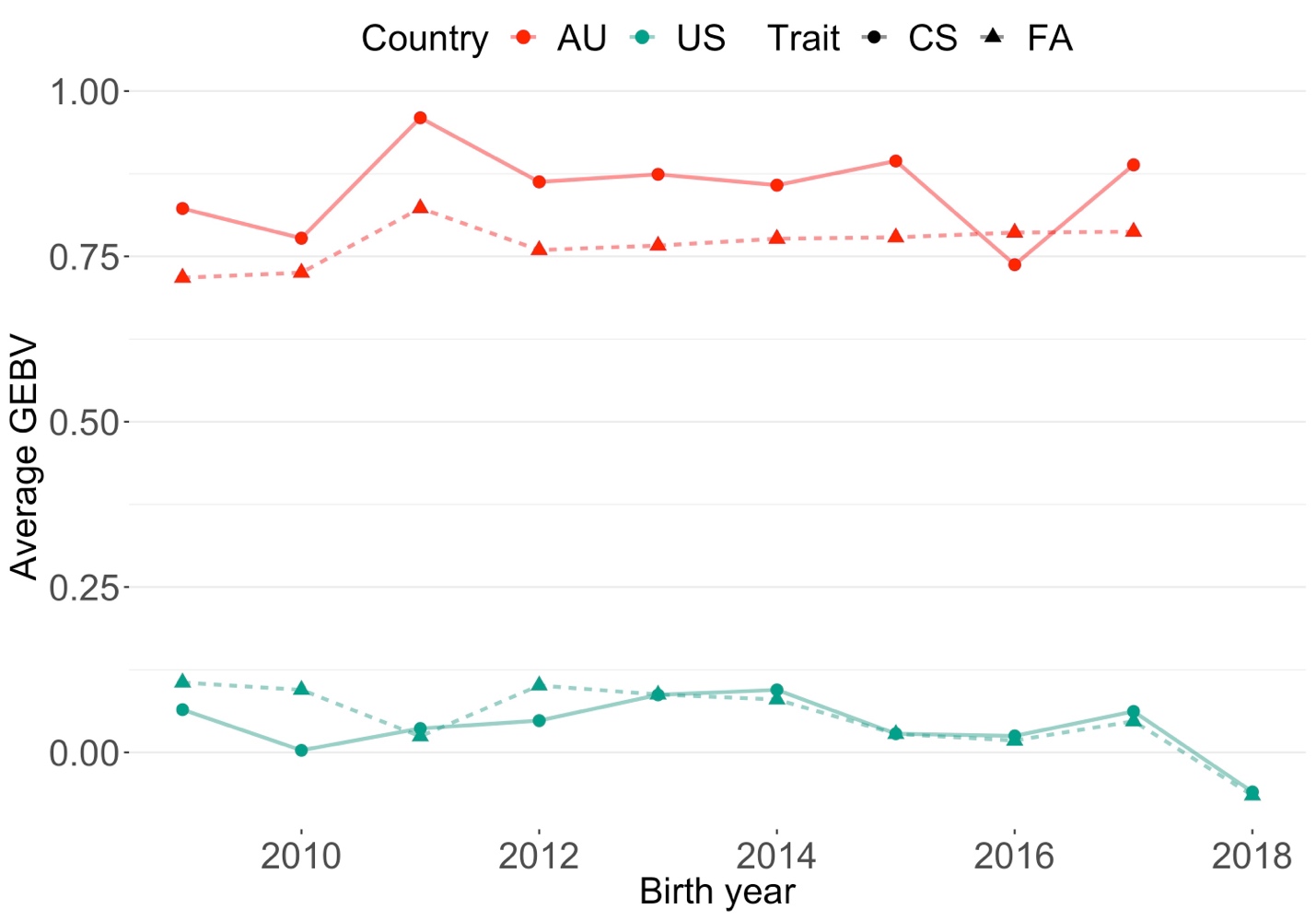


**Figure S6** Genetic trend of foot angle (FA) and claw set (CS) for US and AU separately.

The results come from a single-trait single-country analysis, in which 51,146 genotyped were used for US and 27,041 for AU (only genotyped animals with direct impact on the solution of mixed model equations: phenotyped-genotyped animals and genotyped animals in the pedigree tracing back four generations). Furthermore, in this plot just sires were used (males with at least one progeny), and years with at least 100 individuals.
